# Intrinsic Correlation between Defects, Structure, and Lithium-Ion Transport Kinetics in Epitaxial LiNi_1/3_Mn_1/3_Co_1/3_O_2_ Thin-Film Cathodes

**DOI:** 10.1021/acsami.5c25555

**Published:** 2026-05-18

**Authors:** Blaž Jaklič, Jan Žuntar, Elena Tchernychova, Gregor Kapun, Tina Radošević, Ana Rebeka Kamšek, Robert Dominko, Matjaž Spreitzer

**Affiliations:** † Advanced Materials Department, Jožef Stefan Institute, Jamova Cesta 39, Ljubljana 1000, Slovenia; ‡ Jožef Stefan International Postgraduate School, Jamova Cesta 39, Ljubljana 1000, Slovenia; § 68913 National Institute of Chemistry, Hajdrihova Ulica 19, Ljubljana 1000, Slovenia; ∥ Institute of Metals and Technology, Lepi Pot 11, Ljubljana 1000, Slovenia; ⊥ Faculty of Chemistry and Chemical Technology, University of Ljubljana, Večna Cesta 13, Ljubljana 1000, Slovenia; # Alistore-European Research Institute, CNRS FR 3104, Hub de l’Energie, Rue Baudelocque, Amiens 80039, France

**Keywords:** NMC cathodes, epitaxy, growth mechanism, defects, 4D-STEM, lithium-ion transport

## Abstract

Epitaxial LiNi_1/3_Mn_1/3_Co_1/3_O_2_ (NMC) thin films are prepared via pulsed laser deposition to model fundamental electrochemical behavior and lithium-ion transport kinetics based on different crystallographic orientations and defect types. The observed growth direction and surface termination of NMC thin films are linked to surface energy minimization, primarily via the *(104)* and *(003)* planes. The shortest diffusion path for lithium-ion transport is achieved for a film thickness of ≈15 nm via optimal *(100)*-oriented growth of NMC, indicating selective growth direction of NMC domains. Analysis of interfaces and local crystal structure revealed two predominant types of defects: antiphase boundaries (APBs) and twinned domains, which are strictly related to the symmetry of the layered structure and columnar epitaxial growth of NMC domains. Electrochemical testing vs Li/Li^+^ at charge/discharge rates from C/10 up to 6 C showed that performance is influenced by both the crystallographic orientation of lithium transport pathways and the presence of structural defects. Specifically, *(104)-* and *(* *1̅08)*-oriented NMC thin films with twinned microstructure exhibited stable cycling, delivering specific discharge capacities of 66.2 μA cm^–2^ μm^–1^ (141.2 mAh g^–1^) and 70.2 μA cm^–2^ μm^–1^ (149.4 mAh g^–1^) at C/10, along with apparent lithium diffusion coefficients of 7.45 × 10^–15^ cm^2^ s^–1^ and 7.95 × 10^–15^ cm^2^ s^–1^, respectively. In contrast, *(003)-* and (*1 0 16)*-oriented thin films exhibited lower apparent lithium diffusion coefficients and limited functionality due to less favorable orientations of lithium slabs, higher density of APBs, and unit cell distortion. These factors contribute to a noticeable decline in average discharge voltage at higher discharge rates across all orientations except *(104)*. This approach reveals an intrinsic correlation between the structural properties and electrochemical response of epitaxial NMC thin films and serves as a future guideline toward high-performance NMC cathodes.

## Graphical abstract

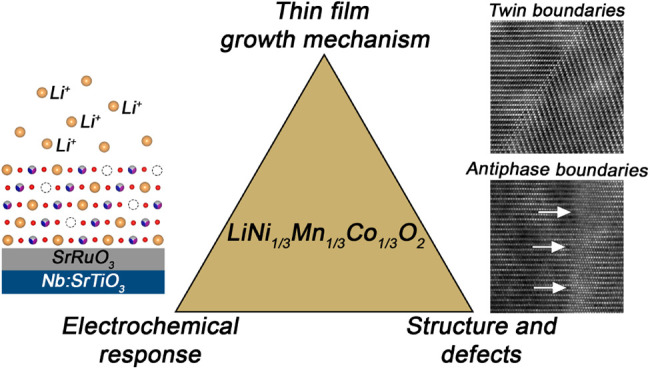

## 1 Introduction

Since the commercialization of the first lithium-ion battery by Sony Corporation in 1991, layered oxide cathodes have been one of the well-studied cathode materials that have attracted considerable technological attention. Decades of research on lithium-ion battery materials resulted in the discovery of LiNi_1‑*x*‑_ *_y_* Mn_*x*_Co_*y*_O_2_ (NMCs), a layered material class with improved energy storage properties.^1^ Replacement of cobalt with nickel increases the specific capacity, while manganese increases thermal stability,^2^ so the properties of NMCs depend on the ratios between transition metal ions.^3^ Density functional theory (DFT) calculations revealed possible migration paths for lithium-ion diffusion in layered oxides that change with lithium concentration, as the ion-hopping mechanism is closely connected to the local vacancy concentration at lithium sites.^4^ Moreover, experimental investigations of the ion conduction mechanism confirmed that lithium transport is much faster in the *ab*-plane compared to ion transport along the *c*-axis.^5^ Nevertheless, experimentally determined activation barriers for ionic transport along the *c*-axis require less energy than expected theoretically.^6^ This apparent discrepancy was addressed by Hasegawa et al., who considered lithium transport along the *c*-axis through structural defects like antiphase boundaries (APBs) and antisite defects in the transition metal layer, which exhibited activation energies similar to those experimentally determined.^7^ On the other hand, the presence of structural defects could initiate irreversible phase transitions, resulting in material degradation after extensive cycling. The most common defects observed in layered oxide cathodes are APBs and twin boundaries (TBs). Direct observation of APBs in the pristine state of the layered NMC crystal structure revealed their formation mechanism, initiated by an edge dislocation.^8^ After extensive cycling, APB length and width enlarged compared to the pristine state and further evolved into an intragranular rock-salt phase, distorting the local crystal structure. A similar structural evolution of APBs in NMC was observed via *in situ* TEM under biasing conditions.^9^ Delithiation of NMC induced the extension of APBs and cation mixing around TBs, which led to structural changes and phase transitions. DFT calculations suggested that the formation energy of APBs decreases in the delithiated structure, while TBs could provide an energetically favorable diffusion path for ion migration, leading to cation intermixing.^9^ Even though APBs and TBs initiate structural disorder that hinders electrochemical performance, it was shown that defect engineering of TBs in layered oxide cathodes could mitigate anisotropic volume changes.^10^ Moreover, our previous study revealed the volume-change compensation mechanism in *(104)*-oriented epitaxial NMC thin films via lattice-plane tilting of the twinned domains.^11^

By using the pulsed laser deposition (PLD) technique, lithium-ion-based cathode thin films can be grown epitaxially without including binders and conductive additives that are commonly used in traditional batteries, so intrinsic material properties can be investigated in PLD-grown thin-film cathodes.^12^ Since the pathways for ionic transport in layered oxide cathodes are preferentially along the *ab*-plane, the orientation of lithium slabs is an important parameter that influences the electrochemical properties. Several groups have been successful in preparing NMC thin-film cathodes by using PLD.^13–20^ Thin films were grown on pure and Au-buffered stainless steel,^16,17^ platinized silicon,^14,15^ sapphire,^17^ and perovskite^13,18–20^ substrates. The quality of deposited thin films can, in fact, be controlled by the growth template, so choosing the right substrate is crucial. NMC thin films grown on stainless steel substrates exhibited poor crystallinity, while growth on Au-buffered stainless steel and platinized silicon substrates resulted in highly textured thin films.^15–17^ On the other hand, epitaxial thin films that are obtained on sapphire^17^ and Nb-doped SrTiO_3_ (Nb:STO)^13,18–20^ single-crystal substrates proved to be useful for manipulating the crystallographic orientation of thin-film cathodes. Even though oxide substrates generally exhibit high resistivity, Nb:STO substrates provide sufficient electronic conductivity for the electrochemical analysis of thin films for battery applications. However, Nb:STO is an n-type semiconductor that causes rectification at the interface with p-type high-voltage cathodes, so the deposition of an additional buffer layer that improves charge transfer is required before NMC thin-film deposition.^21^ The growth of NMC epitaxial thin films is strongly related to the stability of different low-index NMC surfaces. DFT calculations revealed that the most stable NMC 111 surface is *(104),* with a surface energy of 0.76 J/m^2^, followed by the *(003)* surface with a surface energy of 0.89 J/m^2^.,^22^ Calculations predicted that the *(110)* surface undergoes reconstruction to minimize the surface energy via *(104)* facets, while *(102̅)* and *(100)* surfaces exhibit higher surface energies of 1.97 J/m^2^ and 2.27 J/m^2^, respectively.^22^ This directly influences the selective growth direction of NMC domains, which is governed by the minimization of surface energy via *(104)* and *(003)* surfaces. The growth of NMC single crystals via molten-salt-mediated synthesis revealed the importance of the exposed crystalline facets, as increased exposure of *(104)* facets at the expense of *(003)* facets facilitated lithium-ion transport while suppressing oxygen vacancy formation, consequently improving the electrochemical performance and structural stability of NMC single crystals.^23^ In the case of thin films, it is possible to synthesize layered oxide cathodes with different orientations of lithium slabs by manipulating the substrate orientation, directly influencing the solid-state diffusion path of lithium ions from the bulk to the surface. Hirayama et al. performed structural and electrochemical analyses to study the influence of thin-film orientation on electrochemical behavior in epitaxial NMC thin-film cathodes.^18^ Although functional anisotropy in NMC has been revealed, the fundamental correlation between the degree of structural order and defect density with the electrochemical response at different rates of charge and discharge should be systematically investigated, since it is strongly related to the nucleation and growth of the multidomain layered crystal structure.

This study reveals the growth mechanism of epitaxial NMC thin films on different SrRuO_3_/Nb:SrTiO_3_ surfaces, which is primarily related to the minimization of surface energy. Different types of defects (APBs, TBs) are formed as a consequence of the columnar epitaxial growth of the NMC multidomain crystal structure. The possibility of minimizing the diffusion length for lithium-ion transport from the bulk to the surface of the thin films via the growth of *(100)*-oriented NMC on Nb:STO *(211)* is realized for the initial thickness of 15 nm of the thin film; with increasing thickness, less desirable orientations started to dominate. Electrochemical analysis of NMC thin-film cathodes via galvanostatic cycling at different current densities vs Li/Li^+^ revealed the correlation of the crystal orientation and local defect concentration to the general electrochemical response, while cyclic voltammetry measurements provided the apparent diffusion coefficients of lithium in epitaxial NMC thin films. Understanding the defect formation mechanism and the relationship between structural properties and functionality in NMC provides fundamental insight into the material properties, which is essential for the rational design of high-performance cathodes and the development of solid-state thin-film batteries as energy storage systems for a variety of devices such as wireless sensors, detectors, medical implants, and wearable electronics.

## 2 Experimental Methods

### 2.1 Thin-Film Deposition

LiNi_0.33_Mn_0.33_Co_0.33_O_2_ target with 30% lithium excess was prepared in-house by the solid-state synthesis route, as described in previous work.^11^ SrRuO_3_ (99.95%) target was purchased from Beijing Goodwill Metal Technology. NMC thin films were synthesized using a pulsed laser deposition system (TSST, Netherlands) equipped with a 248 nm ultraviolet KrF excimer laser (Coherent COMPex 205) with a 20 ns pulse. Films were grown on 0.5 wt % Nb-doped SrTiO_3_ *(001)*, *(110)*, *(111),* and *(211)* single crystals (Shinkosha, Japan). Before NMC deposition, the SrRuO_3_ film was grown epitaxially on Nb:STO. The optimization of deposition parameters for SRO and NMC is described in our previous work.^11^ The heating and cooling rate was set to 10 °C/min. RHEED patterns were recorded during the growth to monitor surface structure changes.

### 2.2 Structural and Surface Characterization

The θ-2θ patterns, rocking curves, azimuthal φ patterns, reciprocal space maps (RSMs), and X-ray reflectometry (XRR) measurements were collected using an X-ray diffractometer (Empyrean, Malvern PANalytical) with Cu*K*α_1_ radiation (λ = 1.5406 Å). A double-bounce Ge *(220)* hybrid monochromator was used on the incident-beam side. A PIXcel3D detector captured and analyzed the diffracted beam. Rocking curves were measured on symmetric NMC peaks, except for the *(1 0 16)*-oriented NMC, where the *(0 0 12)* plane was chosen for rocking curve analysis due to its higher intensity. In azimuthal φ scans, χ was tilted at an angle to align the Nb:STO *(001)* or *(110)* planes and the NMC *(003)* or *(104)* planes with the X-ray beam, depending on the substrate and thin-film orientations. For RSMs, the sample and the beam were aligned to asymmetric Nb:STO planes before the measurements. The values of the RSM data were converted from angular units to reciprocal space coordinates *Q* (*Q*_*x*_ for the in-plane component, *Q*_*y*_ for the out-of-plane component) using the equations *Q*_*x*_ = *R*(cosω – cos­(2θ – ω)) and *Q*_*y*_ = *R*(sinω + sin­(2θ – ω)), where *R* = 1/2. The results are presented in the form of contour plots of intensity versus *Q*_*x*_ and *Q*_*y*_ in reciprocal lattice units (*r.l.u.*). For X-ray reflectometry measurements, a parallel plate collimator was used on the diffracted side. The thin film thicknesses were estimated from XRR measurements via the Fourier method.

TEM lamella sample preparation was performed by using a FIB Helios Nanolab 650 (Thermo Fisher Scientific, The Netherlands). The TEM lamella sample preparation followed the same procedure as described in our previous work.^11^ The cross-sectional samples of interfaces were examined in the *[010]* zone axis of the NMC thin films by a JEM-ARM200CF probe Cs-corrected scanning transmission electron microscope (STEM) equipped with a cold field emission electron source operated at 80 kV. 4D-STEM data sets were acquired using a MerlinEM pixelated detector (Quantum Detectors, Oxford, U.K.) with a convergence angle of ∼7 mrad. Virtual dark-field images were obtained by integrating intensities using an annular binary mask as a virtual aperture in reciprocal space. The data were preprocessed using the natural logarithm of one plus the signal value to enhance low-intensity features and then analyzed with k-means clustering.^24^ QSTEM software was used to perform (4D-)­STEM simulations with instrumental parameters matching the experimental ones.^25^ Average diffraction patterns were obtained after 10 iterations of each simulation, as the average grid of a 10 × 10 grid of diffraction patterns.

Atomic force microscopy (AFM) was performed with a Veeco Dimension 3100 SPM instrument to study the surface morphology of the films. AFM images were analyzed and edited with WSxM 5.0 software.^26^

### 2.3 Electrochemical Characterization

In order to study the electrochemical response of NMC thin films, NMC|Li liquid electrolyte pouch cells were assembled inside an Ar-atmosphere glovebox and electrochemically analyzed. For the pouch cell assembly step, 110 μm thick lithium foil (FMC Corporation) with a 12 mm diameter was punched and gently brushed with a plastic cylinder to obtain a fresh metal surface. For the separator, 18 mm disks were punched out of 260 μm thick glass fiber paper (Whatman, GF/A glass microfiber). LP40 (1 M LiPF_6_ in EC:DEC 1:1 vol., Sigma-Aldrich) was used as the electrolyte. The pouch cell was made out of polypropylene/polyethylene/polypropylene laminated Al foil, with thin metal foil strips serving as contacts (Al foil for the NMC thin-film electrodes, and Cu foil for lithium anodes).

The electrochemical response of NMC 111 thin-film electrodes was tested under different current densities via a rate testing program on NMC|Li half-cells in the pouch cell configuration. The cells were first slowly charged to 4.2 V vs Li/Li^+^ with a current density of 0.2 μA cm^–2^ and then underwent two discharge–charge cycles in the range of 3.0–4.2 V vs Li/Li^+^ at 0.4 μA cm^–2^ (C/10), ensuring the formation of a stable interface. Afterward, three discharge–charge cycles were performed at current densities of 2 μA cm^–2^, 4 μA cm^–2^, 8 μA cm^–2^, 16 μA cm^–2^, 24 μA cm^–2^ and finished with 2 μA cm^–2^, corresponding to C-rates of C/2, 1 C, 2 C, 4 C, 6 C, and C/2, respectively. For the cyclic voltammetry (CV) measurements, the cells were first slowly charged to 4.2 V and discharged to 3.0 V with a current density of 0.8 μA cm^–2^ (C/5). Afterward, cyclic voltammetry measurements were performed in the potential range from 3.0 V (OCV, if OCV < 3.0 V) to 4.2 V vs Li/Li^+^ with potential sweep rates of 0.05, 0.1, 0.15, 0.2, 0.25, 0.3, 0.35, 0.4, and 0.45 mV s^–1^ for the slower CV measurements and 0.5, 1, 2, 3, 4, and 5 mV s^–1^ for the faster CV measurements.

## 3 Results and Discussion

### 3.1 Influence of Substrate Orientation on the Structural Properties and Morphology of Epitaxial NMC Thin Films

The deposition of NMC 111 thin films on SRO/Nb:STO substrates is studied *in situ* via RHEED (Figure 1a-d) for substrates with *(001)*, *(110)*, *(111)*, and *(211)* out-of-plane orientations. On the basis of RHEED patterns recorded after the annealing process, NMC thin films grow via the Frank-van der Merwe type of growth on *(001)* and *(111)* substrates, as indicated by a streaky RHEED pattern. In comparison, 3D island-type growth is observed on the *(110)* and *(211)* substrates, indicated by transmission spots in the pattern. Additional RHEED patterns of Nb:STO and as-deposited SRO (Figure S1) provide further information about the surface structure during the deposition. All single-crystalline substrates are flat and smooth but vary in their surface structure after SRO deposition. The as-deposited surfaces of SRO on *(001)*, *(111)*, and *(211)* are flat, while SRO grows in an island-like morphology on the *(110)* substrate, influencing the subsequent NMC film growth. AFM images of annealed NMC thin films are shown in Figure 1i-l. NMC thin films are dense and rather smooth for all of the orientations. The smoothest surface is observed for the NMC thin film grown on *the (001)* substrate, with a corresponding RMS surface roughness of 0.36 nm, which increases to 1.02, 1.34, and 1.41 nm when NMC is grown on *(110), (111)*, and *(211)* substrates, respectively. The thicknesses of the SRO bottom electrodes and NMC thin films on different orientations were estimated by X-ray reflectometry (XRR) via the Fourier method to be 35, 34, 35, and 44 nm for SRO and 72, 61, 63, and 75 nm for NMC 111 deposited on *(001)-*, *(110)-*, *(111)-*, and *(211)*-oriented Nb:STO substrates, respectively (Figure S2).

**Figure 1:**
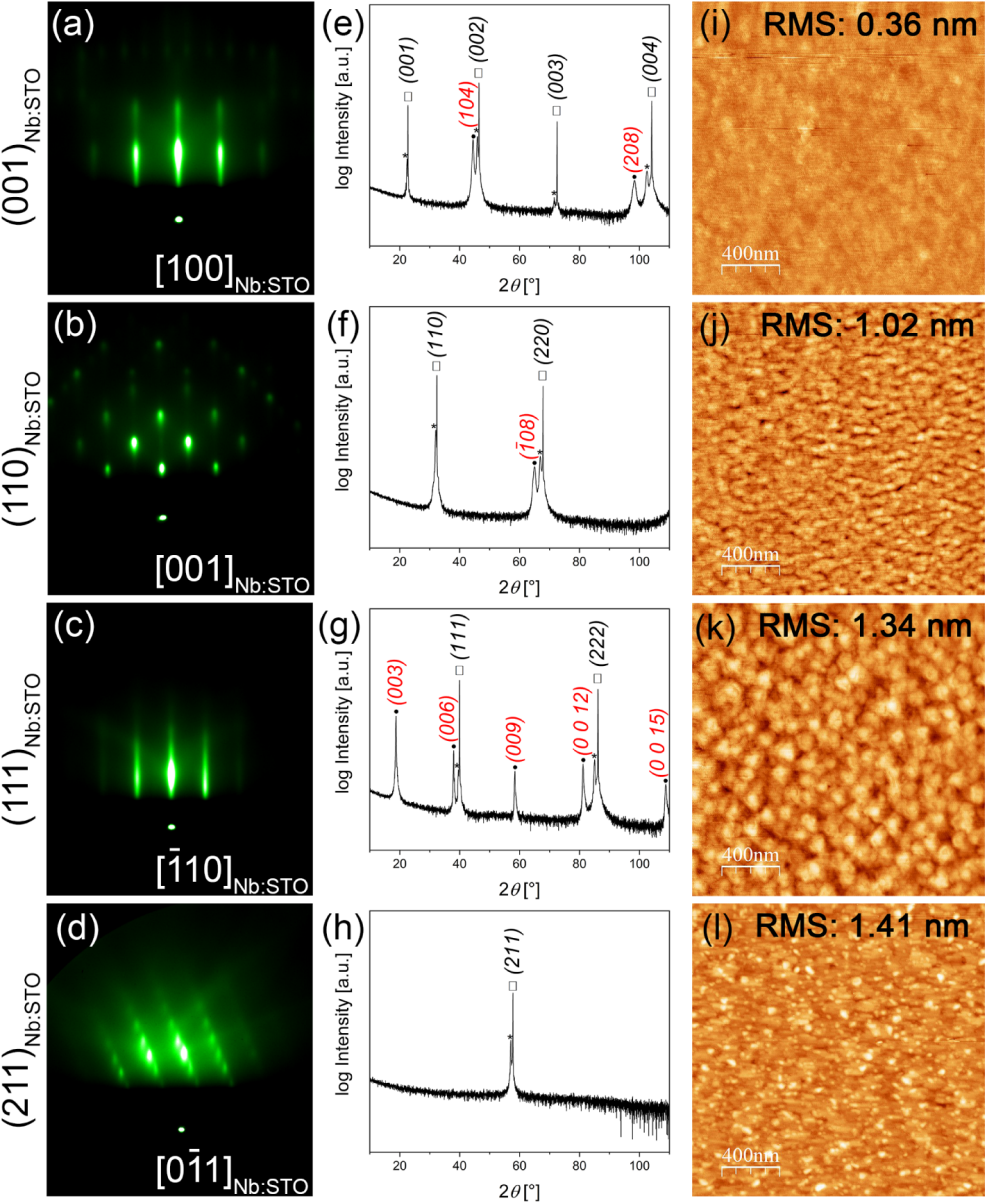
(a-d) RHEED patterns of NMC thin-film surface structures recorded after annealing. (e-h) Out-of-plane XRD patterns of NMC thin films on SrRuO_3_/Nb:SrTiO_3_ substrates. * SrRuO_3_ peaks, square Nb:SrTiO_3_ peaks, bold dot NMC 111 peaks. (i-l) AFM images of pristine NMC thin films after annealing.

The symmetrical out-of-plane X-ray diffraction (XRD) patterns of NMC thin films are presented in Figure 1e-h. The diffraction peaks can be attributed to the Nb:STO substrate (square), the SRO thin-film electrode (*), and the NMC 111 thin-film reflections (bold dot). SRO reflections show the same out-of-plane orientation as the substrate for all corresponding orientations since it has a cubic perovskite crystal structure with a lattice parameter close to Nb:STO. On the other hand, NMC reflections show different out-of-plane orientations depending on the substrate, due to its hexagonal crystal structure, resulting in *(104)*,*(1̅08)*, and *(003)* out-of-plane orientations on Nb:STO *(001)*, *(110),* and *(111)*, respectively. The positions and fwhm values of characteristic NMC *(104)*,*(1̅08)*, and *(003)* out-of-plane reflections (Figure S3) are comparable to the peak positions of polycrystalline NMC 111 ^27^ with differences of 0.09°, 0.72°, and 0.05°, respectively. Based on the hexagonal symmetry of the layered NMC crystal structure, both *(1̅08)* and *(110)* surfaces epitaxially match the SRO/Nb:STO *(110)*, which explains the shift of *(1̅08)* peak positions toward higher angles and the increased fwhm value, since the contribution of the NMC *(110)* reflection cannot be excluded. Various reports agree with such crystallographic alignment of NMC with R3̅*m* symmetry on different SRO/Nb:STO structures,^18,20,28^ but there are no data on NMC growth on *(211)* substrates. The idea behind NMC thin-film growth on the *(211)* substrate is to epitaxially align the substrate lattice with NMC *(100)* planes, which would result in the orientation of lithium slabs perpendicular to the substrate surface and the shortest path for lithium-ion diffusion from bulk to the surface. Since diffraction peaks of the NMC thin film on Nb:STO *(211)* were not observed in a symmetrical XRD scan, additional measurements are necessary to understand the orientation relationship between the substrate and the film. By utilizing fast RSMs on the NMC thin film grown on the *(211)* substrate in the *[0* *1̅* *1]* direction of the substrate (Figure S4), reflections of all layers are visible. The observed NMC thin-film reflections suggest the orientation of NMC to be *(1 0 16)* in the out-of-plane direction, as the peak positions of the film reflections match the R3̅*m* NMC layered structure with this specific crystallographic orientation. Additionally, high-resolution RSM revealed a shift of the in-plane reciprocal direction *Q*_*x*_ of the NMC *(1 0 16)* reflection (Figure S4) from the symmetric line in reciprocal space, which indicates the epitaxial layer tilt of the NMC thin film on the *(211)* substrate, discussed in Supporting Information file and calculated to be ≈ 1°. Inclined epitaxy, commonly observed in the heteroepitaxial growth of semiconductors, is strongly connected to the interface and the lattice mismatch between the substrate and the epitaxial layer, explained by the mismatch at the surface steps of the vicinal substrate and the presence of misfit dislocations.^29,30^ In the case of NMC growth, the inclination of the epitaxial layer could follow the same mechanism as inclined epitaxy in semiconductors due to the large lattice mismatch (Table 1) and increased interface roughness with SRO *(211)*.

**Table 1:** Pseudocubic Lattice Parameters and Lattice Mismatches between the Substrate and NMC Thin Films, as Shown in Figure 4

substrate orientation	*(001)*	*(110)*	*(111)*	*(211)*
a (sub)	5.52 Å	3.91 Å	4.89 Å	6.76 Å
b (sub)	5.52 Å	5.52 Å	5.52 Å	5.52 Å
a (NMC)	5.79 Å	4.08 Å	4.97 Å ***(003)***	7.13 Å ***(100)***
			5.03 Å ***(102̅)***	7.03 Å ***(1 0 16)***
b (NMC)	5.73 Å	5.73 Å ***(1̅08)***	5.73 Å	5.73 Å
		5.79 Å ***(110)***		
a (lattice mismatch)	–4.9%	–4.3%	–1.6% ***(003)***	–5.8% ***(100)***
			–2.9% ***(102̅)***	–4.0% ***(1 0 16)***
b (lattice mismatch)	–3.8%	–3.8% ***(1̅08)***	–3.8%	–3.8%
		–4.9% ***(110)***		

Rocking curves on NMC peaks (Figure 2a-d) reveal the differences in mosaicity between NMC thin films, which are related to different substrate orientations. The slimmest curve is measured for NMC on Nb:STO *(111)* with the corresponding fwhm of 0.09°, which indicates a uniform alignment of NMC *(003)* crystalline planes, while the mosaicity increases when lithium slabs are inclined to the substrate surface, increasing the fwhm value of the rocking curve to 0.24°, 1.27°, and 1.43° for the NMC thin film on *(211)-*, *(001)-,* and *(110)*-oriented substrates, respectively. Moreover, in-plane symmetry relations between the film and the substrate are studied via azimuthal φ scans, shown in Figure 2e-h. NMC grown on the *(001)* substrate (Figure 2e) is thoroughly investigated in our previous work,^11^ where a 4-domain in-plane crystallographic orientation is the consequence of the twinned microstructure. A similar relation is observed in NMC grown on the *(110)* substrate, with a 2-domain in-plane crystallographic orientation and a 90° in-plane tilt of NMC *(003)* planes with respect to the *(001)* substrate planes (Figure 2f). NMC grown on the *(111)* substrate (Figure 2g) aligns well with the substrate in the in-plane direction, which has 3-fold symmetry. A small contribution of NMC *(104)* planes appears in the φ scan with a 60° tilt to the substrate, which indicates a small degree of in-plane twinning. On the other hand, only one peak is observed in the azimuthal φ scan for the NMC grown on the *(211)* substrate (Figure 2h), which suggests the absence of twinning in the *(1 0 16)*-oriented thin film.

**Figure 2:**
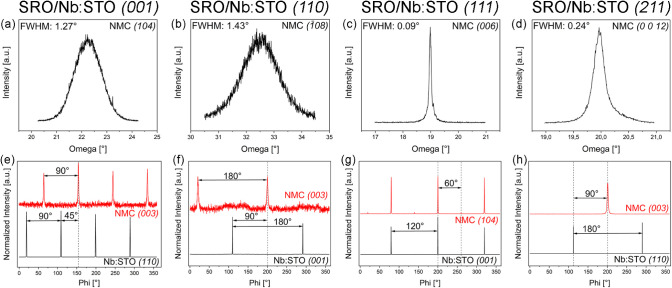
(a-d) Rocking curves and (e-h) azimuthal φ patterns of NMC thin-film reflections on SrRuO_3_/Nb:SrTiO_3_ substrates.

To characterize the degree of strain in SRO and NMC thin films, RSMs are collected around asymmetrical reflections for each orientation. Figure S5 depicts RSMs collected around asymmetrical Nb:STO and SRO reflections, which prove that SRO is strained to the substrate for all orientations. The NMC thin film is proven to be completely relaxed on SRO/Nb:STO *(001)* (Figure 3a), *(110)* (Figure 3b), and *(111)* (Figure 3c) substrates, as the in-plane reciprocal direction *Q*_*x*_ of the substrate and the film are not aligned. Contrary, the reciprocal space map collected around the NMC *(0 0 12)* reflection, grown on SRO/Nb:STO *(211)* (Figure 3d), reveals the alignment of reciprocal lattice points in *Q*_*x*_, which is a result of the inclined epitaxy of the NMC thin film. Since RSMs provide the strain state only in a specific direction, additional RSMs were collected from the crystallographic directions that are in-plane rotated by 90° (Figure S6) with respect to the substrate directions indicated in Figure 3. These additional RSMs proved the strain relaxation of epitaxial NMC thin films, independent of orientation.

**Figure 3:**
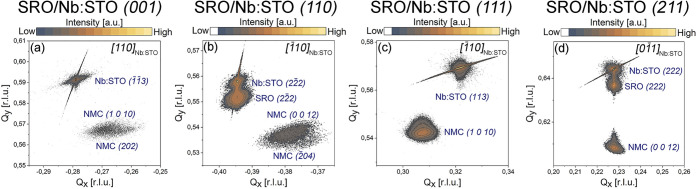
Reciprocal space maps collected around the Nb:STO and NMC reflections of thin films deposited on (a) *(001)-*, (b) *(110)-*, (c) *(111)-,* and (d) *(211)*-oriented substrates.

Figure 4 presents structural models of cross-sections and relaxed lattice planes of NMC thin films grown on SRO/Nb:STO substrates, with corresponding lattice constants and lattice mismatches in different crystallographic directions shown in Table 1. Lattice constants and mismatches are calculated from STO and NMC crystal structures reported in the literature (Table S1). SRO is considered fully strained to the substrate; therefore, lattice constants in the in-plane direction are the same as the substrate, and lattice mismatch is calculated as the difference between the substrate and NMC lattice constants. The lattice mismatch values between the NMC film and the substrate are considered too large to strain the film to the substrate; therefore, the film grows completely relaxed on SRO/Nb:STO substrates. To further clarify the orientation relationship and strain state of the NMC thin film deposited on SRO/Nb:STO *(211)*, a selected area electron diffraction (SAED) pattern was recorded in the *[010]* zone axis of the NMC and *[0* *1̅* *1]* zone axis of the substrate, as shown in Figure S7, including the simulated diffraction patterns of NMC *(1 0 16)* (red dots), NMC *(100)* (blue dots), and SRO/Nb:STO *(211)* (green dots). SAED revealed the in-plane alignment of the NMC *(006)* reflection with the SRO/Nb:STO *(111)* reflections, complementary to the RSM observation of fourth-order diffraction spots in Figure 3d. However, other reflections of the NMC thin film differ in the in-plane spacing compared to the substrate, which proves that the alignment of certain reflections is a matter of epitaxial tilt rather than coherent strain matching of the film to the substrate. By measuring the exact positions of specific thin-film reflections in reciprocal space, hexagonal lattice parameters and unit cell volumes (Table 2) of NMC thin films are calculated using the procedure described in Supporting Information file. The results show slight variations in the lattice parameters between relaxed thin films, while the epitaxially tilted NMC thin film exhibited a significant decrease in the *a* lattice parameter and unit cell volume. This is strongly correlated to the layer tilt of the NMC thin film, which seems to significantly distort the lattice of the layered crystal structure. Nevertheless, it should be noted that these calculations consider a single-phase R3̅*m* NMC layered structure, so structural defects and the presence of other phases could influence the calculated lattice parameters and unit cell volumes.

**Figure 4:**
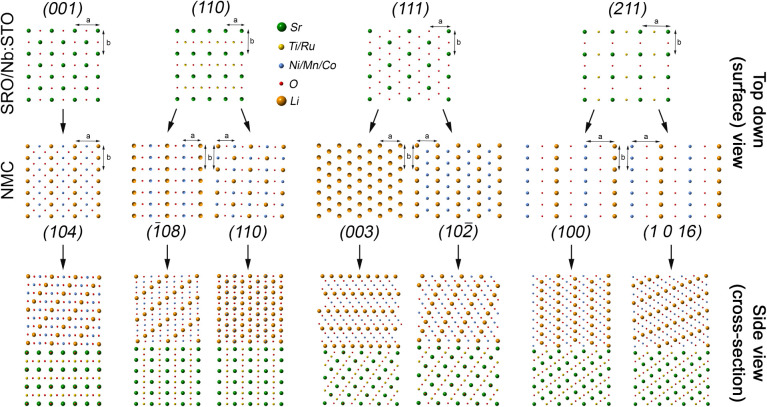
Structural models of SRO/Nb:STO substrate and NMC thin-film surfaces and cross-sections.

**Table 2:** Hexagonal Lattice Parameters and Unit Cell Volumes of Epitaxial NMC Thin Films Deposited on Different Nb:STO/SRO Substrates

Substrate orientation	*a* lattice parameter	*c* lattice parameter	Unit cell volume
*(001)*	2.88 Å	14.23 Å	102.4 Å^3^
*(110)*	2.89 Å	14.05 Å	102.3 Å^3^
*(111)*	2.89 Å	14.21 Å	103.1 Å^3^
*(211)*	2.83 Å	14.22 Å	98.6 Å^3^

To understand the mechanism behind thin-film growth on different SRO/Nb:STO substrates, different surface planes that could grow on each single-crystalline substrate surface should be considered. While the NMC *(104)* plane matches well with the *(001)* surface of the substrate, different growth directions are possible on other substrate orientations since *(110*) substrate surface matches with the NMC *(1̅08)* and *(110*) planes, *the (111)* substrate surface matches with the NMC *(003)* and (102̅) planes and *the (211)* substrate surface matches with the NMC *(1 0 16)* and *(100)* planes, as presented in Figure 4. The lattice parameters of the aforementioned sets of NMC planes are similar, with slight differences in one in-plane direction, leading to smaller mismatches with the substrate for *(1̅08)*, *(003),* and *(1 0 16)* planes, and are therefore considered more likely.

### 3.2 Interfaces and Defects in Epitaxial NMC Thin Films

To correlate and understand the influence of substrate orientation on NMC thin-film growth and local crystal structure, FIB lamellae are cut in the *[010]* NMC zone axis to reveal a layered crystal structure based on the determined in-plane symmetry relations. Cross-sections of NMC/SRO/Nb:STO stacks on different substrate orientations (Figure 5) confirmed the smooth interface between the SRO bottom electrode and NMC thin films on *(001)* and *(111)* Nb:STO, while increased interface and surface roughness were observed on *(110)* and *(211)* Nb:STO, which aligns well with the RHEED patterns recorded during thin-film deposition. STEM micrographs of SRO–NMC interfaces confirmed the suggested orientation of twinned domains (indicated with red arrows) in NMC thin films on Nb:STO *(001)* (Figure 5a) and *(110)* (Figure 5b), which are tilted by ≈ 55° and ≈35° to the substrate surface, respectively. Considering the possible growth orientation of the NMC thin film on SRO/Nb:STO *(110)* (Figure 4), experimental observation of lithium slabs in the Nb:STO [*1̅* *10]* direction proves that NMC is preferentially oriented in the *(1̅08)* direction. In addition, the lithium slabs in NMC thin films on Nb:STO *(111)* are preferentially oriented parallel to the substrate surface (Figure 5c). Minor regions of NMC *(102̅)* are observed experimentally (Figure S8a), while the preferable growth of the thin film follows the *(003)* direction, given the lower surface energy.^22^ This observation is strongly connected to the growth of the NMC thin film on Nb:STO *(211)* (Figure 5d). Closer examination of the NMC–SRO interfaces on Nb:STO *(211)* reveals the *(111)* and *(001)* termination of the SRO layer (Figure S8b), demonstrating increased interface roughness. Based on SRO termination, the NMC thin-film growth follows the same direction as observed and confirmed on *(001)* or *(111)* surfaces, which explains why two out-of-plane orientations are observed at the interface with SRO *(211)*.

**Figure 5:**
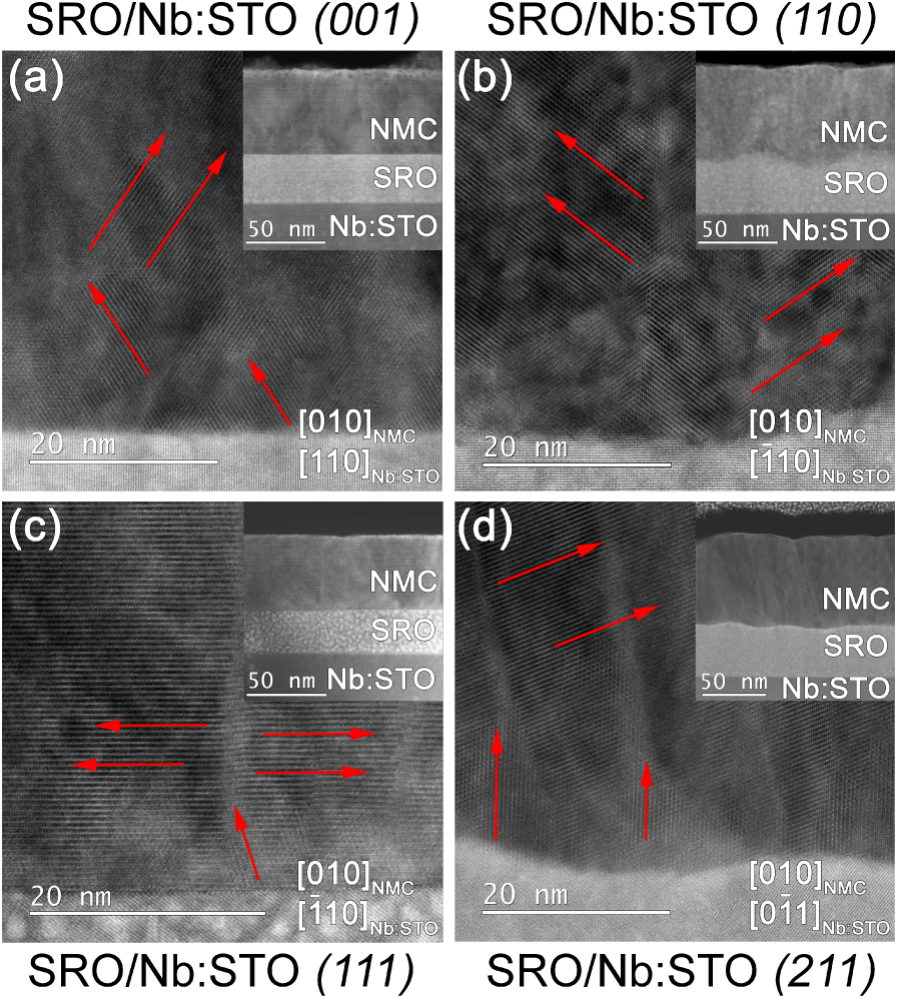
STEM analysis of NMC–SRO interfaces and cross-section inserts of (a) NMC/SRO/Nb:STO *(001)*, (b) NMC/SRO/Nb:STO *(110)*, (c) NMC/SRO/Nb:STO *(111),* and (d) NMC/SRO/Nb:STO *(211)*.

In situ monitoring of the RHEED pattern during NMC thin-film deposition on SRO *(211)* (Figure S9) reveals additional diffraction spots at the early stages of growth, which are indicative of *(100)*-oriented NMC. Direct observation of the surface structure evolution reveals that nucleation and initial thin-film growth on SRO/Nb:STO *(211)* follows the *(100)* and *(1 0 16)* directions, while the latter becomes dominant after 2000 pulses, corresponding to a film thickness of ≈15 nm. Based on local structural observation at the interface, NMC thin-film growth is strongly influenced by the SRO bottom electrode, which is preferentially *(001)*- and *(111)*-terminated and governed by the minimization of surface energy. This leads to the preferential growth of NMC thin films in the *(104)* and *(003)* directions, which epitaxially match the *(001)* and *(111)* SRO surfaces, respectively.

Surface termination and growth direction of NMC thin film directly affect the formation of structural defects. Figure 6a reveals the symmetric *(104)* out-of-plane oriented domains in the NMC thin film, twinned across the *(1̅08)* at an angle α ≈ 35°, perpendicular to the substrate surface. A reversed twin boundary defect is observed in the NMC *(1̅08)* thin film (Figure 6b), where two domains are twinned across the *(104)* plane at an angle β ≈ 55°, also described as 90° – α. This type of defect between domains is isostructural to the ones observed in NMC thin films grown on *(111)* and *(211)* substrates. For example, two out-of-plane orientations that grow in the *(100)* and *(1 0 16)* directions (Figure 6c) are symmetric across the *(104)* plane, which resembles previously discussed domain boundary defects. Interestingly, as the thin-film growth progresses, no domain boundaries symmetric across the *(1̅08)* plane are observed, which could be related to thermodynamic factors (minimization of the total surface energy leading to the preferential growth direction). It should be noted that the crystallographic notation of twinning planes is merely shown to clarify similarities between domain boundaries, rather than being the exact twinning plane. Except for the well-defined coherent twin boundary in Figure 6b, the boundaries between the domains are usually interconnected in a 3D framework and are therefore related to the initial orientation of domains that grow into each other as the film growth progresses. Apart from the domain boundaries, STEM analysis revealed the presence of antiphase boundaries (APBs) that are observed in NMC *(1 0 16)* (Figure 6d) and NMC *(003)* (Figure 6e), where they could extend throughout the film and reach the surface of the NMC thin film, as shown in Figure S10. We assume that APB-type defects form as a consequence of the large lattice mismatch between SRO and NMC, compensated through multiple domain rotations and dislocations, which initiate APB formation at the surface steps of the SRO substrate. Besides the formation of APBs via edge dislocations at the interface, domains could merge and form APB-type defects during thin-film growth.

**Figure 6:**
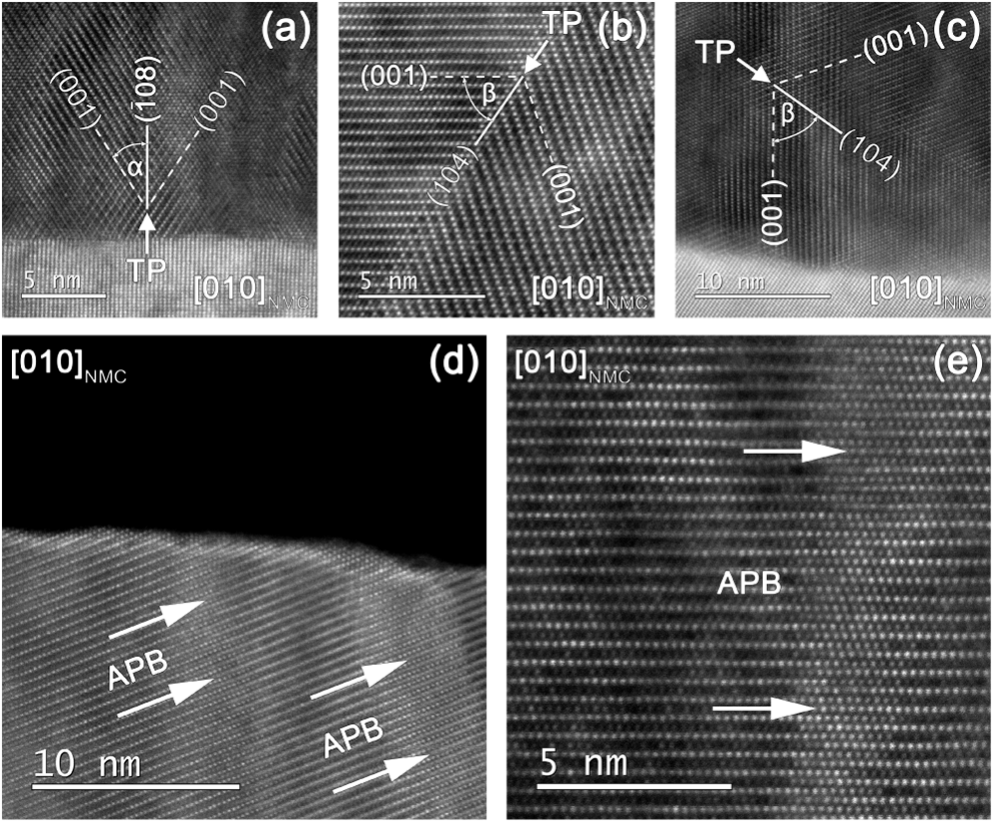
STEM analysis of structural defects in NMC thin films. (a) Twinned domains in NMC *(104)* thin film. (b) Coherent twin boundary in NMC *(1̅08)* thin film. (c) *(100)-* and *(1 0 16)*-oriented domains, symmetric across *the (104)* plane. (d) Antiphase boundaries in NMC *(1 0 16)* thin film. (e) Antiphase boundary in NMC *(003)* thin film.

Additionally, the differences in the distribution and low-angle rotations of domains in NMC thin films were investigated with 4D-STEM. A representative area, consisting of both the SRO layer and the NMC thin film, was recorded for each sample, as shown in the virtual dark-field images in Figure 7a. Clustering was used to segment the imaged areas into parts with a specific diffraction signal. The k-means algorithm forms clusters of diffraction patterns based on the pixel-wise distances between them, with each pattern belonging to one cluster. The algorithm partitioned the 256 × 256 grid of recorded diffraction patterns into a number of clusters, chosen so that one cluster could be attributed to the SRO layer, and the rest exhibited distinct diffraction signals associated with the NMC thin film. The clustering results for all samples are included in Figure 7b. The average diffraction patterns of all clusters were inspected and interpreted in terms of the local crystal structure. While some parts of the film were entirely in a zero-order Laue zone (ZOLZ), making their diffraction disks simple to discern, other parts exhibited somewhat smeared and overlapping disks. In the figure, we include examples of this contrast and highlight parts of the film with a distinct diffraction signal that is not immediately obvious from the virtual dark-field images. Figure S11a presents the average diffraction patterns for clusters associated with the SRO layer (indicated with gray color), where the disk distribution shows a different symmetry, as expected from other experimental results. Averaged patterns of other clusters are included in Figure S11b, mostly belonging to the NMC thin film and, in some cases, to an amorphous carbon layer on top of the thin film (indicated with black color). The difference between the cluster average diffraction patterns can most often be explained by small rotations of domains making up the NMC thin film. To support this argument, Figure S11c includes the results of numerical simulations using a model system—a spherical NMC crystallite with an approximate diameter of 6 nm. Its HAADF-STEM image is provided to confirm that the model is imaged in the same way as the atomically resolved STEM results presented elsewhere in the manuscript. The average diffraction pattern, obtained by simulating a 4D-STEM data set acquisition, includes a similar distribution of Bragg disks next to the ZOLZ as in experimental data. When rotating the model by 1° or 2° away from the zone axis, the intensity distribution of the Bragg disks shifts, and their center of mass is located away from the ZOLZ. Furthermore, the Bragg disks forming a first-order Laue zone are also not centered. While higher-order disks are weak in the experimental data, this kind of shift can still be seen in, e.g., NMC on SRO/Nb:STO *(110)* in Figure 7b. By comparing the k-means clustering analysis of epitaxial NMC thin films (Figure 7b), noticeable differences in domain rotations are observed. First, NMC on SRO/Nb:STO *(001)* features multiple clusters that are somewhat smaller in size and more randomly distributed compared to the NMC on SRO/Nb:STO *(110)*, as a consequence of the in-plane rotational symmetry with higher order, as determined by XRD (Figure 3e–f). The relatively large fwhm values observed for both out-of-plane (Figure 2a–b) and in-plane misorientation distributions (Figure S12) suggest the presence of multiple rotational variants among twinned domains in NMC on SRO/Nb:STO *(001)* and *(110)*. In contrast, for NMC on SRO/Nb:STO *(111)* and *(211)*, domain rotations are more pronounced in the in-plane direction, as indicated by significantly larger fwhm values of the in-plane misorientation distributions (Figure S12) relative to those of the out-of-plane misorientation distributions (Figure 2c-d). Additionally, 4D-STEM analysis proved low-angle rotations between domains in the NMC on SRO/Nb:STO *(111)*, whereas less distinction between clusters is observed in NMC on SRO/Nb:STO *(211),* featuring uniform rotation of domains at the interface with SRO, closely aligning with the nucleation of multiple out-of-plane orientations of NMC domains on SRO/Nb:STO *(211)*, as presented in HAADF-STEM micrographs (Figure 5d). As the growth becomes selective, domains begin to rotate away from the zone axis, which could initiate the formation of APBs between domains. Nevertheless, 4D-STEM analysis indicates the columnar epitaxial growth of NMC domains that leads to defect formation due to misalignment between adjacent columns across all orientations, which may significantly influence the electrochemical response.

**Figure 7:**
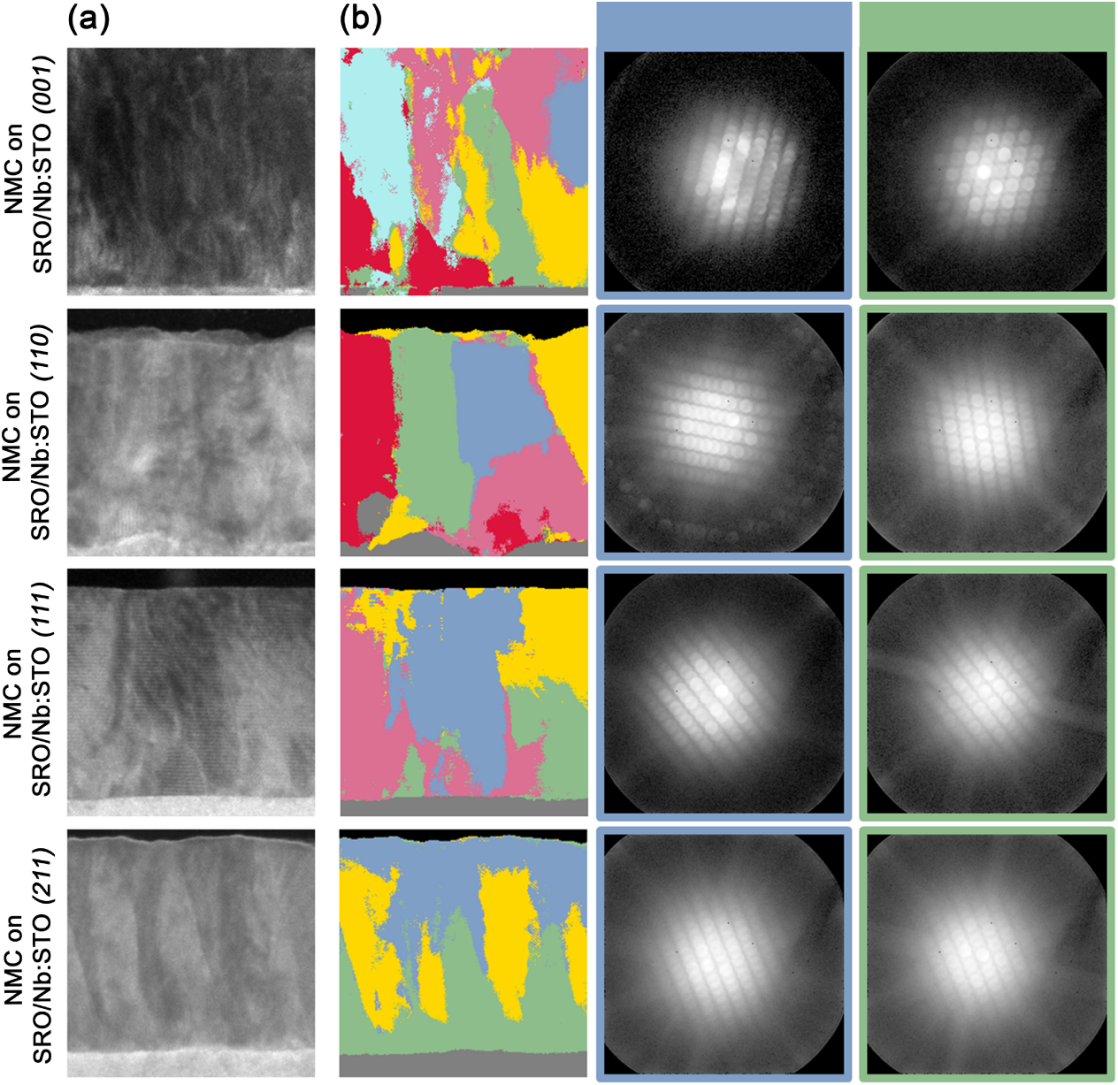
4D-STEM analysis of epitaxial NMC thin films on SRO/Nb:STO substrates. (a) Virtual dark-field images of the imaged area. Sizes of the imaged area from top to bottom: 60 nm, 73 nm, 91 nm, and 84 nm. (b) K-means clustering of the recorded diffraction patterns with color-coded labels (left) and selected cluster average diffraction patterns (right) with a border of the same color as their label.

### 3.3 Intrinsic Electrochemical Properties of Epitaxial NMC Thin Films

The manipulation of the crystal orientation in NMC epitaxial films has been realized by altering substrate orientation, followed by the analysis of the crystal structure and local defects, which is crucial for understanding and investigating the intrinsic functional properties of NMC. The charging and discharging behavior of NMC thin films was tested at different rates of charge and discharge, ranging from C/10 to 6 C, in the potential range from 3.0 to 4.2 V vs Li/Li^+^, as presented in Figure S13. The capacity of NMC thin films is presented in specific volumetric units and calculated from XRR-determined thicknesses. Since the unit cell volume differs in epitaxially grown NMC thin films, the theoretical density is calculated from NMC unit cell volumes (Table 2) via the procedure described in the Supporting Information file. The obtained theoretical density is then used for conversion from volumetric to gravimetric specific capacity.

The NMC *(104)* reached an initial specific discharge capacity of 66.2 μA cm^–2^ μm^–1^ at C/10, corresponding to 141.2 mAh g^–1^. An increased rate of charge/discharge resulted in a gradual loss of capacity, reaching a specific discharge capacity of 38.0 μA cm^–2^ μm^–1^ (81.0 mAh g^–1^) at 6 C (Figure 8a), as observed in the recent study of NMC *(104)* thin films.^11^ A similar capacity fade at higher rates is observed for NMC *(1̅08)*, where the initial specific discharge capacity reached 70.2 μA cm^–2^ μm^–1^ (149.4 mAh g^–1^) at C/10 and faded to 47.2 μA cm^–2^ μm^–1^ (100.4 mAh g^–1^) at 6 C. Both orientations with twinned domains realized stable cycling up to 6 C, with slightly higher specific discharge capacities for NMC *(1̅08)*. This could be related to the increased surface area and more uniform distribution of domains in NMC *(1̅08)*, consequently forming fewer defects that influence lithium-ion transport. However, the fading of the average voltage at higher rates is observed only for NMC *(1̅08)*, while the NMC *(104)* discharge voltage remains stable up to 6 C (Figure 8b). This aligns well with the work of Hirayama et al., as the voltage fade was observed for NMC (1̅08) and NMC *(003)* thin films cycled up to 4.5 V.^18^ On the other hand, NMC *(003)* reached a lower initial specific discharge capacity of 50.3 μA cm^–2^ μm^–1^ (107.9 mAh g^–1^) at C/10 (Figure 8a), while the fading of specific capacity was more pronounced at higher rates, reaching only 7.3 μA cm^–2^ μm^–1^ (15.7 mAh g^–1^) and an average discharge voltage of 3.38 V at 6 C (Figure 8b). Significant overpotential is also observed in NMC *(1 0 16)*, where the fading of the average discharge voltage is the most severe. The specific discharge capacity of NMC *(1 0 16)* is comparable to NMC *(003)*, reaching a specific capacity of 53.8 μA cm^–2^ μm^–1^ (110.5 mAh g^–1^) in the first discharge cycle at C/10 (Figure 8a) that faded to 6.5 μA cm^–2^ μm^–1^ (13.3 mAh g^–1^) with an average discharge voltage of 3.29 V at 6 C (Figure 8b). The poor electrochemical properties of NMC *(003)* and NMC *(1 0 16)* are in the first place related to the crystallographic orientation of the lithium planes, which are oriented parallel to the surface in NMC *(003)* and at an angle of ≈19° to the surface in NMC *(1 0 16)*. Considering lithium-ion transport in NMC *(003)*, ion migration paths along the lithium layers are strictly limited to in-plane movement. Nevertheless, after the charge and discharge cycle at C/10, a reasonable amount of lithium ions is extracted and inserted into the NMC *(003)*, which suggests that lithium ions migrate not only along the lithium layers but also through structural defects, as described in the work of Hasegawa et al., which considers lithium-ion movement through APBs and antisite defects in the transition metal layer.^7^ Besides ion movement through defects, regions of *(102̅)*-oriented domains were observed in STEM, which could allow faster ion transport to the surface of the thin film due to shorter diffusion lengths. Due to large differences in polarization between NMC thin films, it seems that the orientation of lithium slabs influences the degree of overpotential due to kinetic factors during lithium-ion cycling. As the angle between the path for ionic transport in the NMC crystal and the electric field direction is not parallel, this could lead to charge separation and significant polarization at higher rates of charge and discharge. This effect is nicely shown by probing charge distribution in the local nanodomains of radially aligned and randomly oriented grains in polycrystalline NMC.^31^ Different arrangements of the grains in NMC cathodes exhibited similar initial discharge capacities but a noticeable increase in cell polarization for the randomly oriented grains, attributed to the less ordered charge distribution. In addition to the orientation of the lithium slabs, the increased concentration of APBs and the presence of lattice distortion within the thin films may also play a significant role in limiting the functional properties of epitaxial NMC thin-film cathodes. The poor electrochemical performance observed for both *(003)-* and *(1 0 16)*-oriented NMC thin films, therefore, suggests that the functional response cannot be attributed solely to crystallographic orientation.

**Figure 8:**
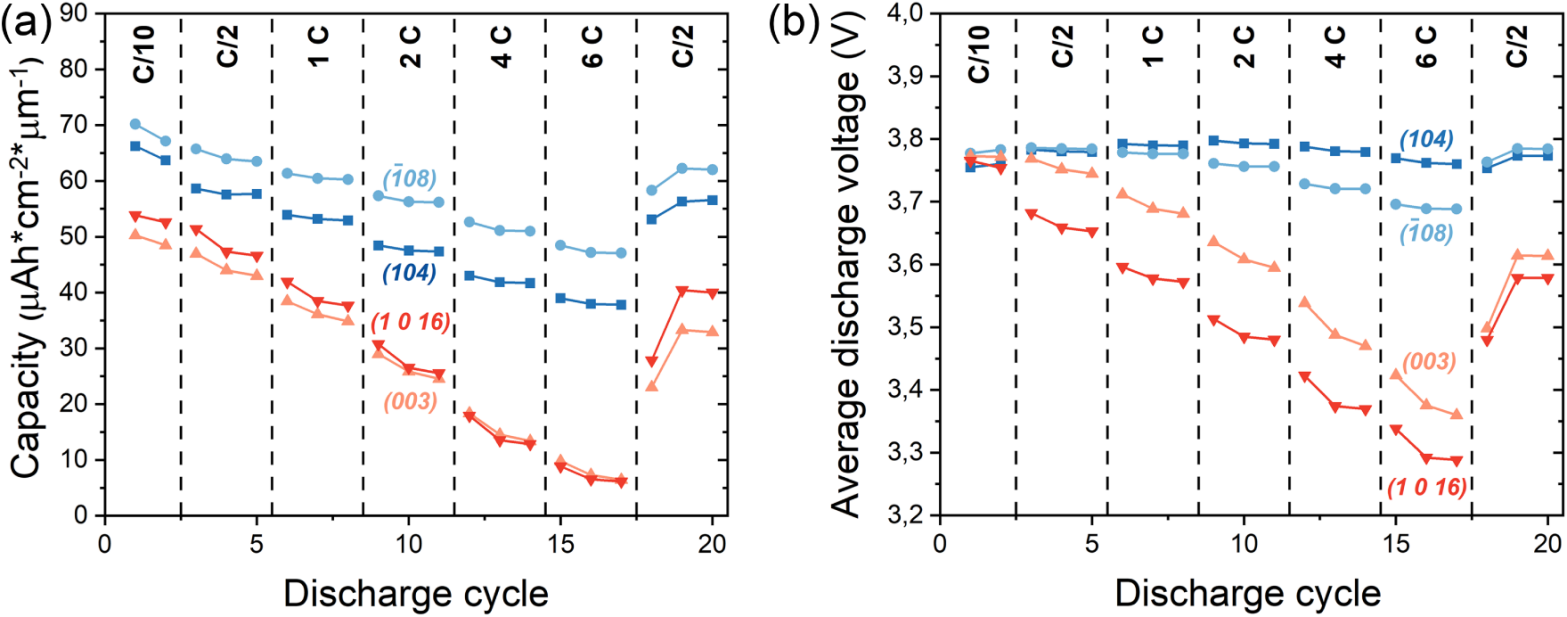
(a) Specific discharge capacity and (b) average discharge voltage comparison of epitaxial NMC thin films cycled vs Li/Li^+^ at current densities of 0.4 μA cm^–2^ (C/10), 2 μA cm^–2^ (C/2), 4 μA cm^–2^ (1 C), 8 μA cm^–2^ (2 C), 16 μA cm^–2^ (4 C), and 24 μA cm^–2^ (6 C).

Additionally, cyclic voltammetry was performed at slower (from 0.05 mV s^–1^ to 0.45 mV s^–1^, as shown in Figure 9a-d) and faster (from 0.5 mV s^–1^ to 5 mV s^–1^, as shown in Figure 9e-h) potential scan rates. Cyclic voltammograms obtained at slower potential scan rates display well-defined cathodic and anodic peaks across all orientations, corresponding to the process of lithium-ion insertion/deinsertion, coupled to the redox reactions of nickel ions.^11^ To support kinetic arguments and quantify lithium-ion transport kinetics, the apparent chemical diffusion coefficient of lithium in epitaxial NMC thin films is obtained via the Randles–Ševčik equation, as described in the Supporting information file. Since the Randles–Ševčik equation assumes semi-infinite linear diffusion, the peak current of the cyclic voltammograms should exhibit a linear correlation with ν^^1^/2^, whereas a linear correlation with ν indicates capacitive-controlled processes. The logarithmic correlation of the peak current with the potential scan rate for the NMC thin-film cathode (Figure S14) reveals the transition from capacitive-controlled behavior toward diffusion-controlled behavior at potential scan rates above 0.5 mV s^–1^. Due to the capacitive contribution at slower rates, which is a consequence of finite lithium-ion diffusion in the NMC thin-film cathode, the apparent diffusion coefficient of lithium is calculated from cyclic voltammograms obtained at higher rates via linearly fitted curves, shown in Figure S15. As observed in Figure 9g and h, higher potential sweep rates significantly distort the peaks in the cyclic voltammograms of NMC *(003)* and NMC *(1 0 16)* thin films, so peak currents at higher sweep rates no longer follow a linear correlation and are therefore excluded from the calculation of the apparent diffusion coefficient. This coincides with the significant polarization observed in the galvanostatic cycling of NMC *(003)* and NMC *(1 0 16)* thin films at higher rates of charge and discharge.

**Figure 9:**
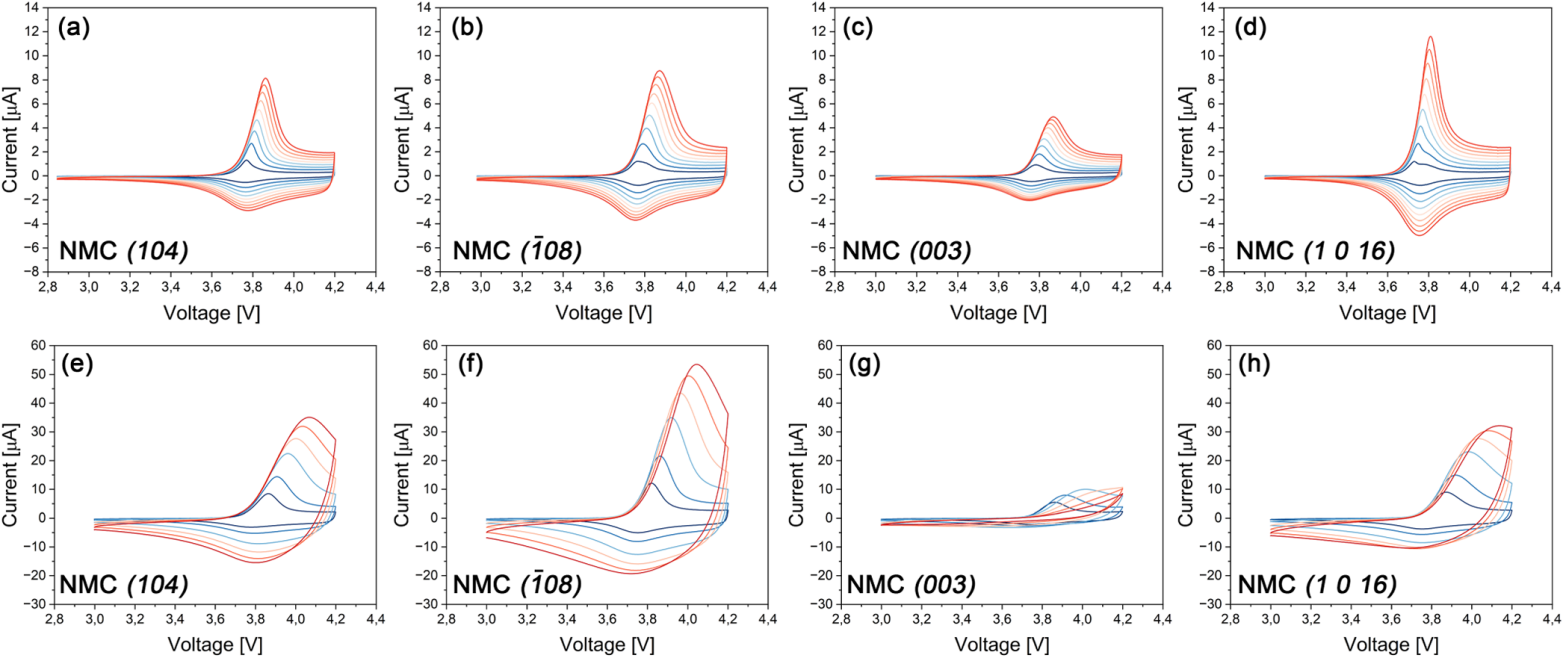
Cyclic voltammograms of epitaxial NMC thin films, cycled vs Li/Li^+^ at potential scan rates (a-d) from 0.05 mV s^–1^ to 0.45 mV s^–1^ and (e-h) from 0.5 mV s^–1^ to 5 mV s^–1^.

The calculated apparent diffusion coefficients for epitaxial NMC thin films during charge and discharge are shown in Table 3. Apparent lithium diffusion values are higher for the delithiation process across all orientations, which is consistent with the CV trends observed for polycrystalline NMC 111.^32^ By comparing the limiting apparent diffusion coefficients (lithiation), the highest values of 7.45 × 10^–15^ cm^2^ s^–1^ and 7.95 × 10^–15^ cm^2^ s^–1^ were obtained by NMC *(104)* and NMC *(1̅08)*, followed by NMC *(1 0 16)* with a value of 3.66 × 10^–15^ cm^2^ s^–1^ and finally NMC *(003)* with an order of magnitude lower apparent diffusion coefficient of 2.90 × 10^–16^ cm^2^ s^–1^, due to the unfavorable crystallographic orientation for lithium-ion transport. It seems that the values of *D̃_Li_* obtained in thin films are significantly lower compared to the polycrystalline NMC electrodes,^32^ due to the significantly higher contact area between the polycrystalline electrode and the electrolyte, which is not taken into account. Nonetheless, the calculated values of *D̃_Li_* across different epitaxial thin films are consistent with the galvanostatic cycling measurements, further confirming the correlation between crystallographic orientation and lithium-ion transport kinetics.

**Table 3:** Apparent Diffusion Coefficients of Lithium, Obtained from Cyclic Voltammograms in the Potential Scan Rates from 0.5 mV s^–1^ to 5 mV s^–1^ for the Lithiation and Delithiation Process in Epitaxial NMC Thin Films

	D̃*_Li_* (cm^2^ s^–1^)
NMC *(104)* delithiation	3.27 × 10^–14^
NMC *(104)* lithiation	7.45 × 10^–15^
NMC *(1̅08)* delithiation	6.49 × 10^–14^
NMC *(1̅08)* lithiation	7.95 × 10^–15^
NMC *(003)* delithiation	4.38 × 10^–15^
NMC *(003)* lithiation	2.90 × 10^–16^
NMC *(1 0 16)* delithiation	3.11 × 10^–14^
NMC *(1 0 16)* lithiation	3.66 × 10^–15^

## 4 Conclusions

Altering the orientation of single-crystalline substrates used for epitaxial growth of NMC thin-film cathodes proved to be an effective approach to investigate the intrinsic functional properties of layered cathodes. Different growth modes of pulsed laser-deposited epitaxial NMC thin films are closely connected to the minimization of surface energy, consequently producing two major defects that were observed on an atomic scale: anti-phase boundaries and twin domains. STEM analysis of NMC thin films grown on SRO/Nb:STO *(001)* and *(110)* revealed the presence of symmetrically twinned domains with R3̅*m* symmetry that are interconnected to each other in a 3D framework. In contrast, APBs were the major structural defect observed in thin films grown on SRO/Nb:STO *(111)* and *(211)*. To minimize the diffusion path for lithium-ion transport along the *ab*-plane toward the surface, NMC thin film is grown in the *(100)* direction on SRO/Nb:STO *(211)*. After an initial thickness of 15 nm, the growth of less desirable orientations starts to dominate as a consequence of surface energy minimization. 4D-STEM analysis provided insight into low-angle rotations within domains and their distribution in epitaxial NMC thin films. Electrochemical testing of the epitaxial thin films illustrated the influence of crystal orientation and defect formation on the electrochemical response. *(104)-* and *(1̅08)*-oriented NMC thin films with twin-domain defects exhibited stable cycling with a gradual fade in specific discharge capacity at higher rates of charge and discharge. On the other hand, *(003)-* and *(1 0 16)*-oriented NMC thin films with an increased concentration of APB defects achieved lower discharge capacities at 0.1 C, while increasing the rate to 6 C resulted in a severe loss of specific discharge capacity and an increase in overpotential, as a result of the unfavorable crystallographic orientation for lithium-ion transport. Additionally, calculation of apparent lithium diffusion coefficients via the Randles–Ševčik equation supported this argument, as *(003)*-oriented NMC exhibited the lowest value among all thin-film orientations. The manipulation of lithium slab orientation on different substrate surfaces revealed the growth mechanism and lithium-ion transport kinetics through different surfaces and defects, which significantly affects functionality. In order to improve the functionality of epitaxial thin-film cathodes with layered structures, the growth of *(104)*-oriented thin films with increased thickness and surface area, while minimizing structural defects that inhibit lithium-ion transport along the *ab*-plane should be considered. To follow up, exploring the growth of NMC thin-film cathodes on alternative substrates or different bottom electrodes to engineer strain states offers a compelling strategy for enhancing their electrochemical performance even further.

## Supplementary Material



## Notes

RHEED patterns of Nb:STO, SRO, and NMC surface structures; X-ray reflectometry patterns; enlarged diffraction peaks of characteristic NMC reflections; fast RSM of *(1 0 16)-*oriented NMC thin film; RSMs of SRO and NMC thin films; selected area electron diffraction pattern of NMC/SRO/Nb:STO *(211)*; equations and parameters for determining layer tilt, lattice parameters, and unit cell volumes of NMC thin films; STEM micrographs of NMC–SRO interfaces; RHEED monitoring of the NMC surface structure during growth on SRO *(211);* STEM micrograph of APB defects; additional cluster average diffraction patterns and numerical 4D-STEM simulations of the model NMC system, in-plane misorientation distributions of NMC domains; galvanostatic cycling curves of NMC thin films cycled vs Li/Li^+^; equations for converting volumetric specific capacity to gravimetric specific capacity; logarithmic correlation between CV-peak current and potential scan rate; square root of scan rate vs CV-peak current correlation; equations and fit parameters for the determination of the apparent chemical diffusion coefficient of lithium (PDF)
