# Supplementary material for: Intrinsic Correlation between Defects, Structure, and Lithium-Ion Transport Kinetics in Epitaxial LiNi1/3Mn1/3Co1/3O2 Thin-Film Cathodes
Source: ACS Appl Mater Interfaces. 2026 May 18;18(21):29832–44. doi: 10.1021/acsami.5c25555 (PMC13244368; doi:10.1021/acsami.5c25555)
Supplement: Supplementary file 1 [file am5c25555_si_001.pdf]

Supporting information

Intrinsic correlation between defects, structure and lithium-ion transport kinetics in epitaxial $LiNi_{1/3}Mn_{1/3}Co_{1/3}O_2$ thin film cathodes

Blaž Jaklič [1,2,], Jan Žuntar [1,2], Elena Tchernychova [3,4], Gregor Kapun [3], Tina Radošević [1], Ana Rebeka Kamšek [3,4], Robert Dominko [3,5,6], Matjaž Spreitzer [1,*]*

[1]Advanced Materials Department, Jožef Stefan Institute, Jamova cesta 39, 1000 Ljubljana, Slovenia

[2]Jožef Stefan International Postgraduate School, Jamova cesta 39, 1000 Ljubljana, Slovenia

[3]National Institute of Chemistry, Hajdrihova ulica 19, 1000 Ljubljana, Slovenia

[4]Institute of Metals and Technology, Lepi pot 11, 1000 Ljubljana, Slovenia

[5]Faculty of Chemistry and Chemical Technology, University of Ljubljana, Večna cesta 13, 1000 Ljubljana, Slovenia

[6]Alistore-European Research Institute, CNRS FR 3104, Hub de l'Energie, Rue Baudelocque, 80039, Amiens, France

Corresponding Authors

*Blaž Jaklič – Email: blaz.jaklic@ijs.si

*Matjaž Spreitzer – Email: matjaz.spreitzer@ijs.si

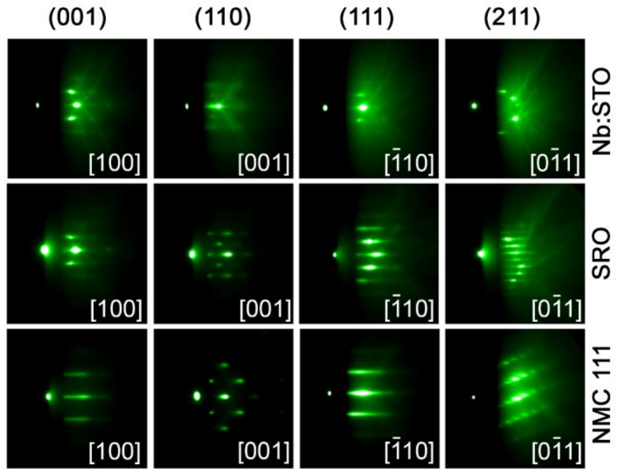

Figure S1. RHEED patterns of pristine Nb:STO substrates, as-deposited SRO bottom electrodes and annealed NMC thin films. All crystallographic notations indicate the direction and orientation of the substrate.

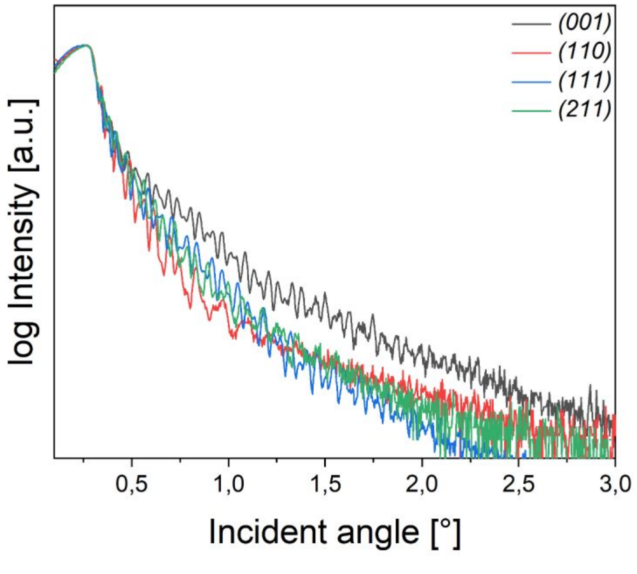

Figure S2. X-ray reflectometry patterns of NMC thin films on $SrRuO_3$/Nb:$SrTiO_3$ substrates with different orientations.

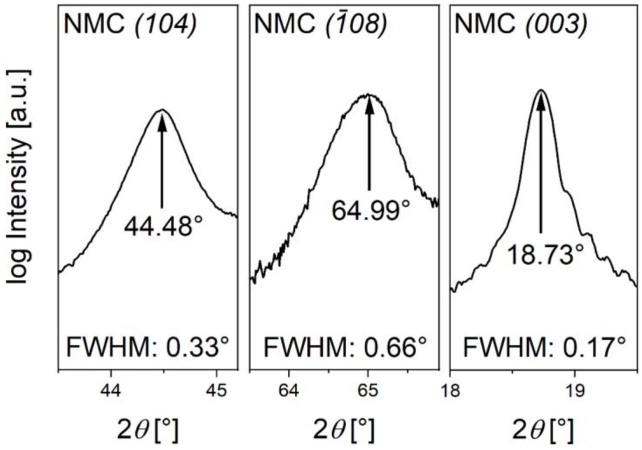

Figure S3. Enlarged diffraction peaks of characteristic NMC out-of-plane reflections.

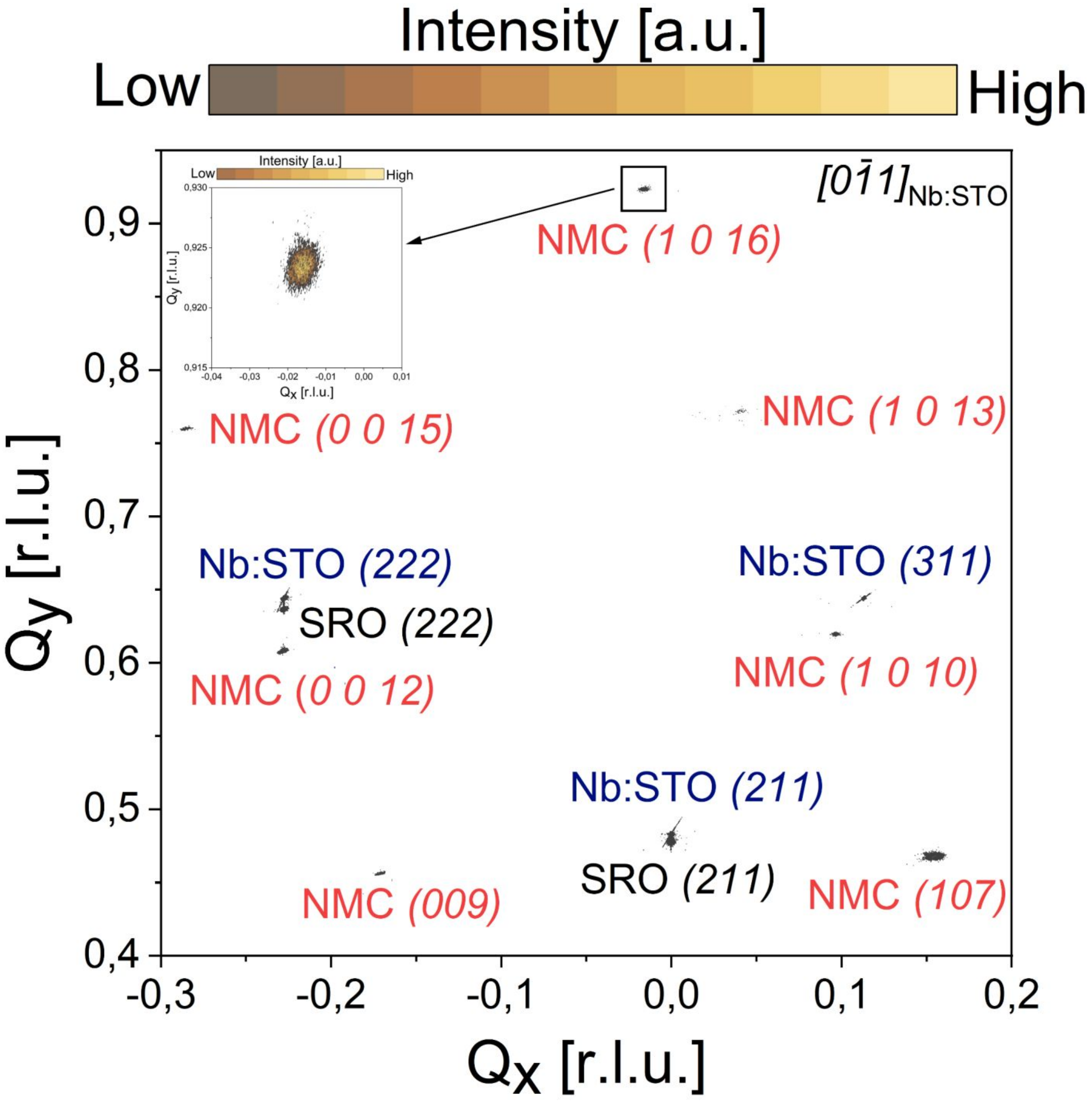

Figure S4. Fast reciprocal space map of *(1 0 16)* oriented NMC thin film.

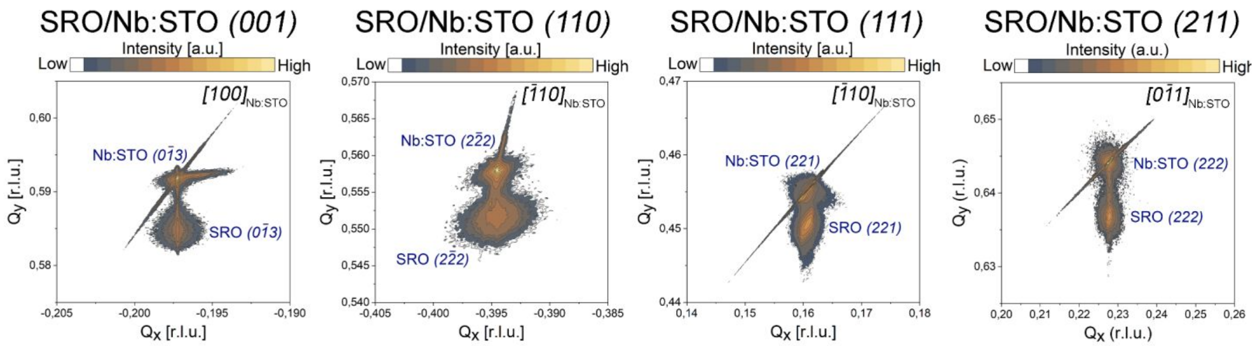

Figure S5. Reciprocal space maps of different out-of-plane orientations collected around asymmetric SRO and Nb:STO reflections.

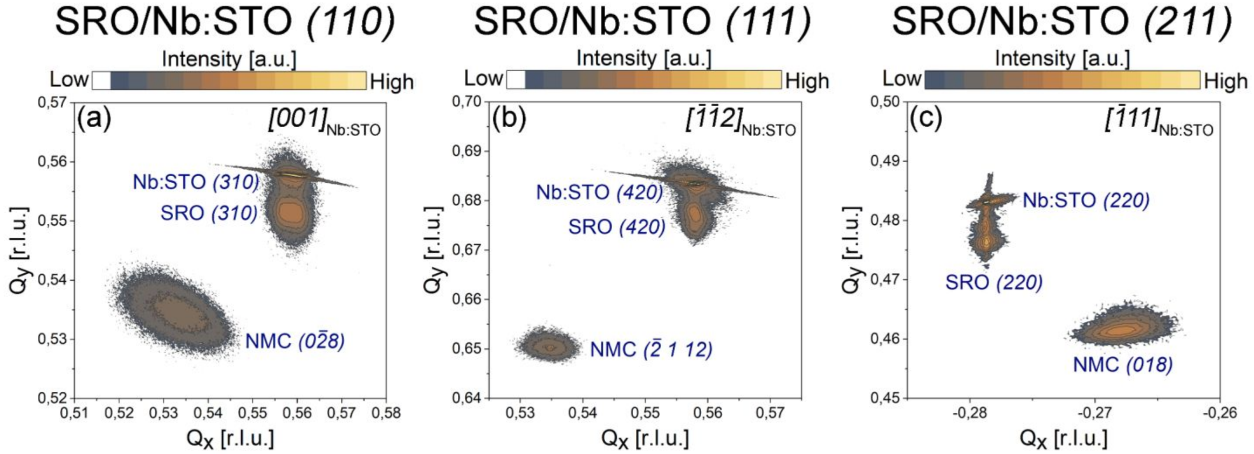

Figure S6. Reciprocal space maps of asymmetric NMC and Nb:STO reflections, collected from the other crystallographic directions of the substrate.

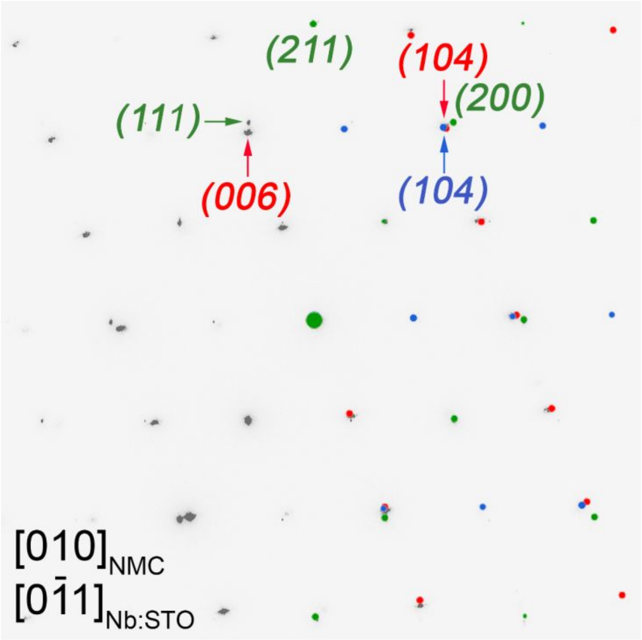

Figure S7. Selected area electron diffraction pattern of NMC/SRO/Nb:STO *(211)*, including simulated diffraction patterns of NMC *(1 0 16)* in red colour, NMC *(100)* in blue colour and SRO/Nb:STO *(211)* substrate in green colour.

To determine the tilt between the substrate and NMC 111 thin film, **Equation S1** is used to calculate the angle of epitaxial tilt α between substrate normal and *(1 0 16)* planes of the thin film layer.

$$\tan\alpha = \frac{Q_{x(1\,0\,16)}}{Q_{y(1\,0\,16)}} \quad (S1)$$

X-Rays are aligned to the substrate before RSM measurement, therefore $Q_x = 0$ for the Nb:STO *(211)* reciprocal lattice point. Since Q_x and Q_y values of *(1 0 16)* plane are determined via RSM, angle of epitaxial tilt α is calculated with **Equation S2**.

$$\alpha = \tan^{-1}\frac{Q_{x(1\,0\,16)}}{Q_{y(1\,0\,16)}} = \tan^{-1}\frac{0.016136}{0.923531} = 1.00097°(S2)$$

Table S1. Relaxed lattice parameters of polycrystalline STO and NMC 111 crystal structures, reported in the literature.

	STO[1]	NMC 111[2]
Space group	$Pm\bar{3}m$	$R\bar{3}m$
a	3.905 Å	2.8663 Å
b	3.905 Å	2.8663 Å
c	3.905 Å	14.262 Å
α	90°	90°
β	90°	90°
γ	90°	120°

(1) Meyer, G. M.; Nelmes, R. J.; Hutton, J. *Ferroelectrics*, **1978**, *21*, 461–462.
(2) Zahnow, J.; Bernges, T.; Wagner, A.; Bohn, N.; Binder, J. R.; Zeier, W. G.; Elm, M. T.; Janek, J. *ACS Applied Energy Materials*, **2021**, *4*, 1335–1345.

To calculate the unit cell parameters from the RSMs, **Equation S3** which correlates d-spacing and lattice parameters in hexagonal unit cell was used:

$$\frac{1}{d^2} = \frac{4}{3}\left(\frac{h^2 + hk + k^2}{a^2}\right) + \frac{l^2}{c^2}$$

Based on **Equation S3**, *a* and *c* lattice parameters of NMC thin films were calculated from the system of two equations, depending on the measured NMC thin film reflections via RSM. **Table**

S2 displays the reflections and equations used for the correct determination of unit cell parameters in epitaxial NMC thin films. Unit cell volume was calculated from **Equation S4**:

$$V_{NMC} = \frac{\sqrt{3}}{2} a^2 c (S4)$$

Table S2. Substrate/thin film reflections and corresponding equations, used for the determination of NMC unit cell parameters.

Substrate orientation	Substrate alignment plane	Thin film reflection 1	Thin film reflection 2	Equation 1	Equation 2
(001)	$(\overline{11}3)$	*(104)*	*(107)*	$\frac{1}{d^2_{(104)}} = \frac{4}{3a^2} + \frac{16}{c^2}$	$\frac{1}{d^2_{(107)}} = \frac{4}{3a^2} + \frac{49}{c^2}$
(110)	*(310)*	$(\bar{1}08)$	$(0\bar{2}8)$	$\frac{1}{d^2_{(\bar{1}08)}} = \frac{4}{3a^2} + \frac{64}{c^2}$	$\frac{1}{d^2_{(0\bar{2}8)}} = \frac{16}{3a^2} + \frac{64}{c^2}$
(111)	*(113)*	*(006)*	*(1 0 10)*	$\frac{1}{d^2_{(006)}} = \frac{36}{c^2}$	$\frac{1}{d^2_{(1\,0\,10)}} = \frac{4}{3a^2} + \frac{100}{c^2}$
(211)	*(222)*	*(009)*	*(107)*	$\frac{1}{d^2_{(009)}} = \frac{81}{c^2}$	$\frac{1}{d^2_{(107)}} = \frac{4}{3a^2} + \frac{49}{c^2}$

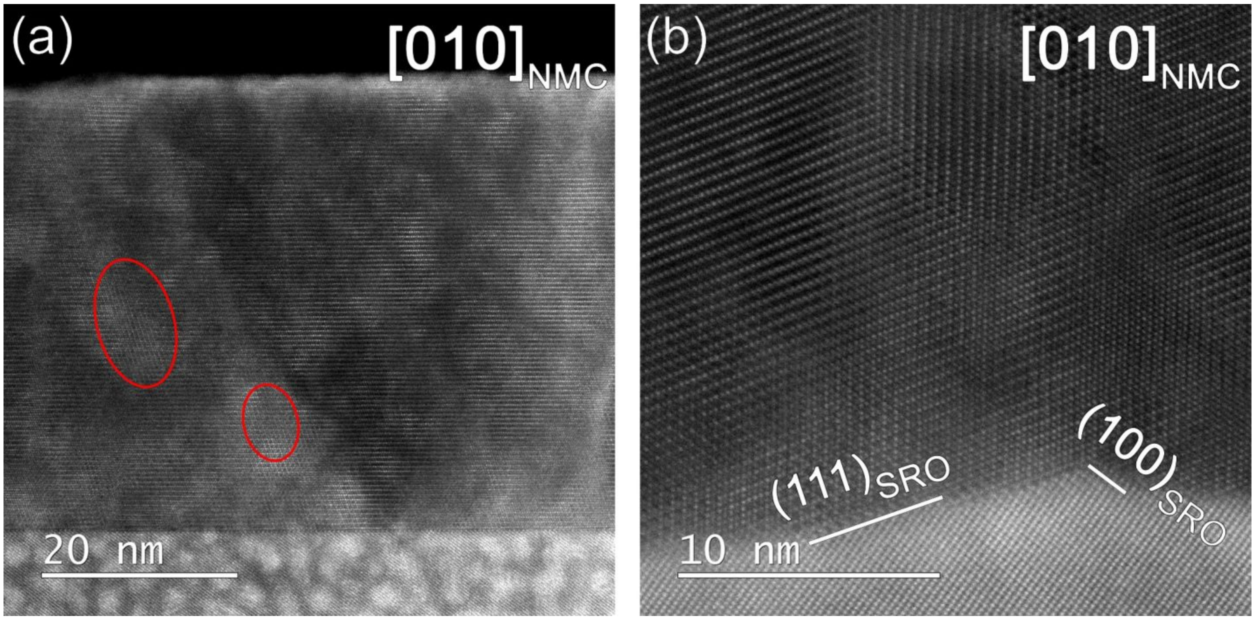

Figure S8. STEM analysis of NMC/SRO interfaces. (a) Marked regions of *(10$\bar{2}$)* oriented NMC, grown on *(111)* SRO surfaces; NMC/SRO interface on (c) *(211)* oriented substrate.

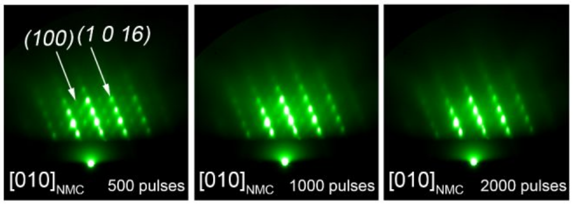

Figure S9. Monitoring the surface structure with RHEED during NMC thin film growth on SRO/Nb:STO *(211)* substrate.

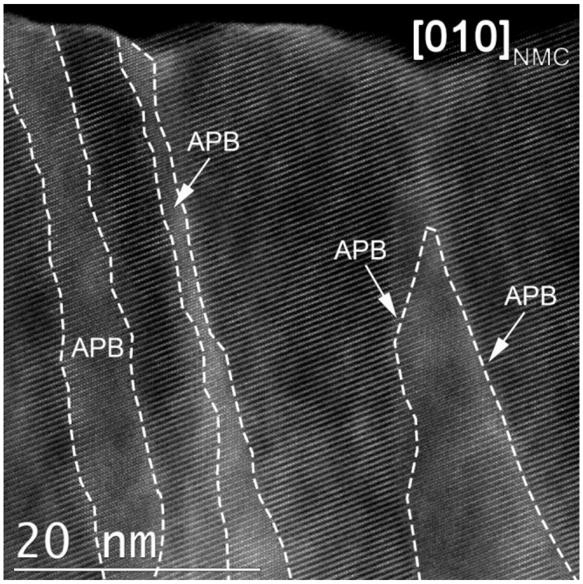

Figure S10. STEM micrograph of anti-phase boundary defects in *(1 0 16)* oriented NMC thin films, indicated with white streaky lines.

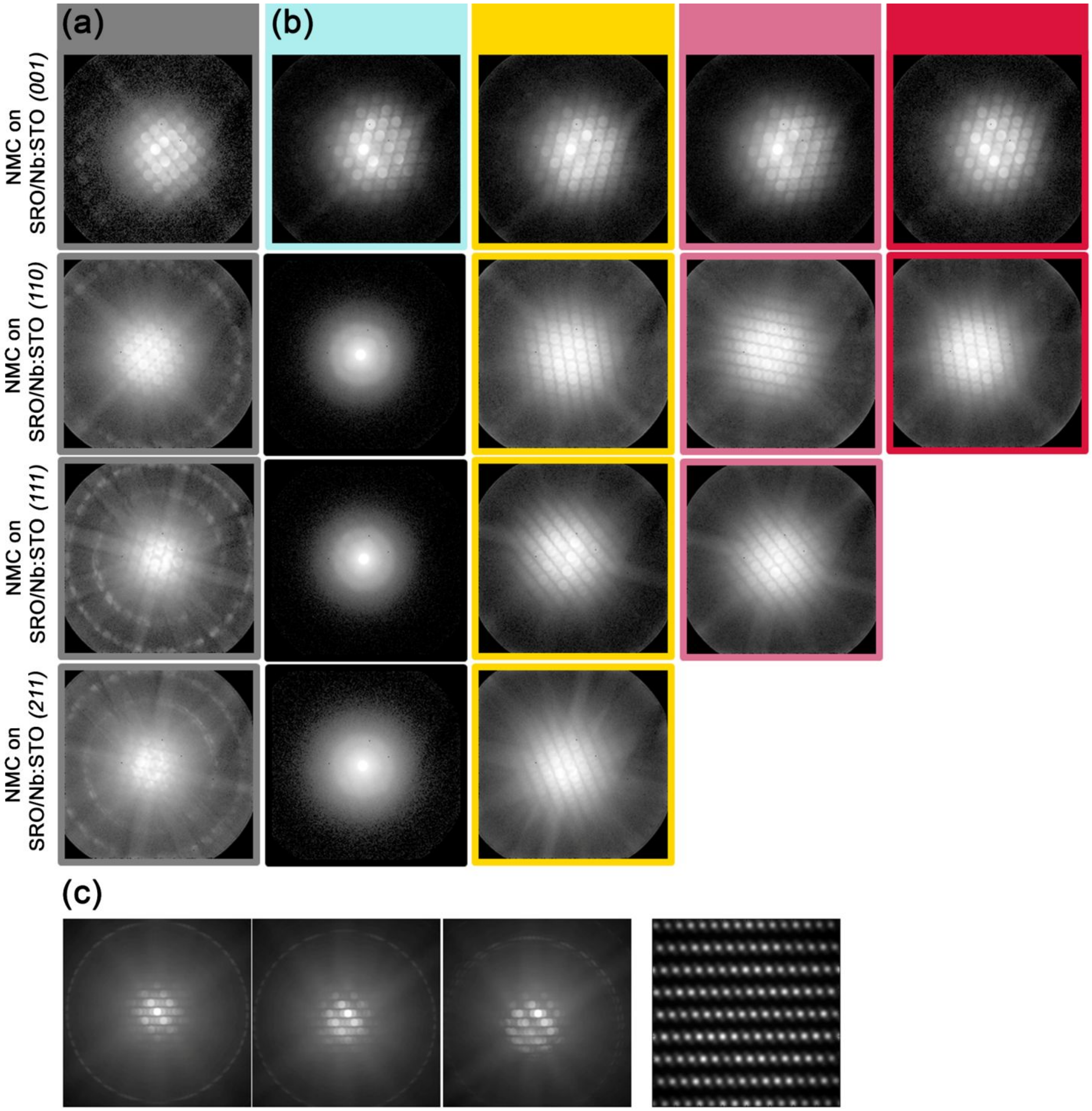

Figure S11. (a,b) Additional cluster average diffraction patterns following k-means clustering for epitaxial NMC thin films on SRO/Nb:STO substrates with different orientations. The patterns in (a) are associated with the substrate (grey color), and the ones in (b) are associated with the NMC thin film, and in two cases, an amorphous carbon (black color) on top of it, as evidenced by the halo signal. (c) Numerical (4D-)STEM simulations of a model NMC system. The simulated

average diffraction patterns are, from left to right, matching a low-index zone axis, and tilted 1° and 2° away from that zone axis. An HAADF-STEM image is shown for the context, with the size of the imaged area being 4 nm x 4 nm.

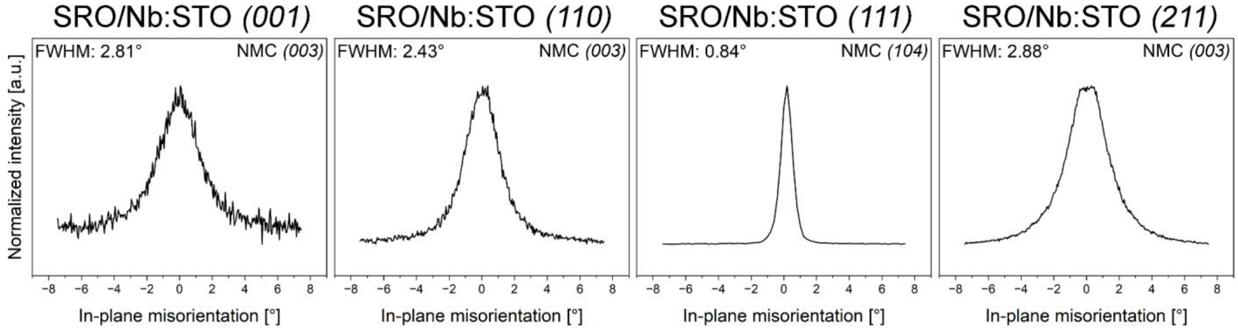

Figure S12. In-plane misorientation distributions of NMC domains, determined via azimuthal φ measurements of NMC thin film reflections on $SrRuO_3$/Nb:$SrTiO_3$ substrates.

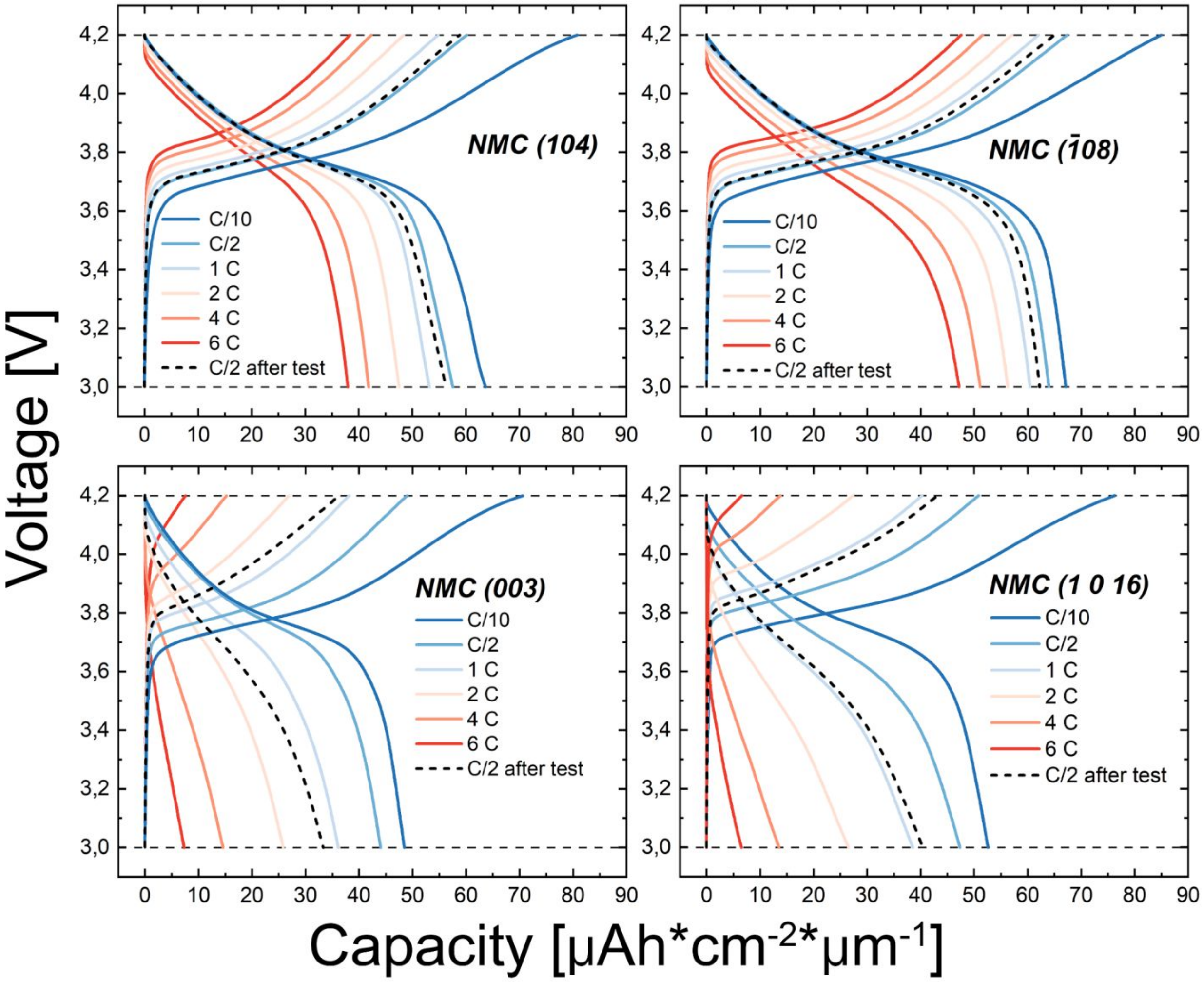

Figure S13. Galvanostatic cycling curves comparison of epitaxial NMC thin films cycled vs. Li/Li^+ at current densities of 0.4 µAcm^{-2} (C/10), 2 µAcm^{-2} (C/2), 4 µAcm^{-2} (1 C), 8 µAcm^{-2} (2 C), 16 µAcm^{-2} (4 C) and 24 µAcm^{-2} (6 C).

To convert the volumetric specific capacity to gravimetric specific capacity, theoretical density is calculated with **Equation S5**, where Z = 3 (formula units per unit cell), $M_{NMC} = 96.46$ g mol^{-1} (molar mass of NMC 111), $N = 6.022 \times 10^{23}$ mol^{-1} and V_{NMC} is unit cell volume of NMC 111. Gravimetric capacity is then converted from volumetric capacity via **Equation S6**.

$$\rho_{NMC} = \frac{Z\, M_{NMC}}{N_A\, V_{NMC}} (S5)$$

$$Gravimetric\ Capacity_{NMC} = \frac{Volumetric\ Capacity_{NMC}}{\rho_{NMC}} (S6)$$

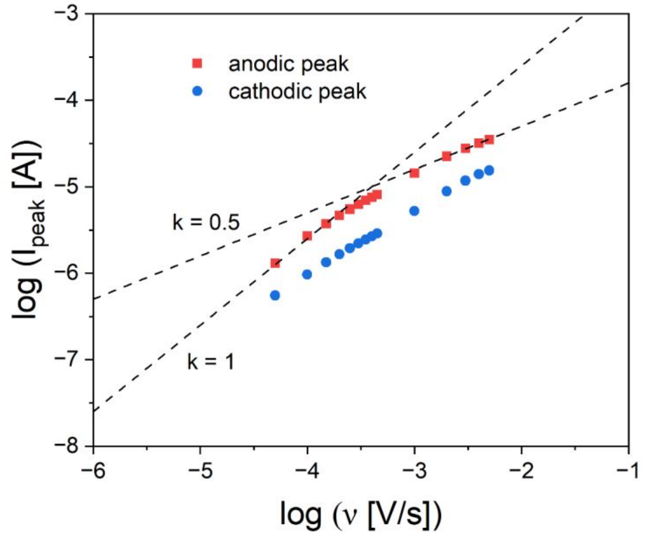

Figure S14. Logarithmic correlation between peak current and potential scan rate of epitaxial NMC *(104)* thin film, obtained from cyclic voltammograms, which indicates diffusion-controlled (k = 0.5) or capacitive-controlled (k = 1) behaviour.

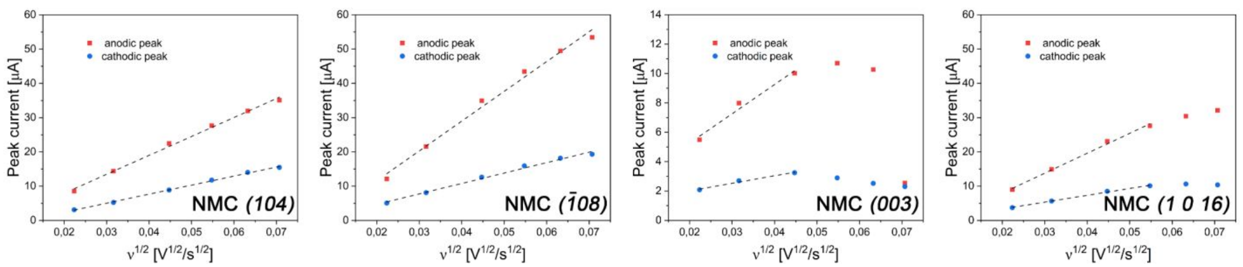

Figure S15. Square root of potential scan rate dependence of the peak current for the epitaxial NMC thin films, obtained from cyclic voltammograms at potential scan rates from 0.5 mV/s to 5 mV/s.

To calculate the apparent chemical diffusion coefficients of lithium from cyclic voltammograms, Randles – Ševčik equation (**Equation S7**) was used, where I_{peak} is a peak current, n is the charge-transfer number of lithium, A is the active electrode area, $\tilde{D}_{Li}$ is the apparent chemical diffusion coefficient, ν is a potential scan rate and C is a concentration of lithium in the thin film electrode:

$$I_{peak} = 2.69 \times 10^5\, n^{3/2}\, A\, \tilde{D}_{Li}^{1/2}\, \nu^{1/2}$$

The apparent chemical diffusion coefficients are then calculated via **Equation S8**, where S is a slope of the linear fitted curve, shown in **Figure S15**, which correlates I_{peak} and $\nu^{1/2}$:

$$\tilde{D}_{Li} = \left(\frac{S}{2.69 \times 10^5\, n^{3/2}\, A\, C}\right)^2$$

Table S3. Slope values of I_{peak} vs. $\nu^{1/2}$ and corresponding R^2 values, obtained from the linear fitted curves, as shown in **Figure S15**.

	S (Slope of $I_{peak}/\nu^{1/2}$)	R^2
NMC *(104)* delithiation	5.53×10^{-4}	0.99571
NMC *(104)* lithiation	2.64×10^{-4}	0.99796
NMC *($\bar{1}$08)* delithiation	8.69×10^{-4}	0.98967
NMC *($\bar{1}$08)* lithiation	3.04×10^{-4}	0.99087
NMC *(003)* delithiation	1.99×10^{-4}	0.97531
NMC *(003)* lithiation	5.13×10^{-5}	0.98316
NMC *(1 0 16)* delithiation	5.81×10^{-4}	0.99519

NMC *(1 0 16)* lithiation	1.99×10^{-4}	0.99629
